# Effect of dimethyl fumarate on mitochondrial metabolism in a pediatric porcine model of asphyxia-induced in-hospital cardiac arrest

**DOI:** 10.1038/s41598-024-64317-9

**Published:** 2024-06-15

**Authors:** Sarah Piel, Meagan J. McManus, Kristina N. Heye, Forrest Beaulieu, Hossein Fazelinia, Joanna I. Janowska, Bryce MacTurk, Jonathan Starr, Hunter Gaudio, Nisha Patel, Marco M. Hefti, Martin E. Smalley, Jordan N. Hook, Neha V. Kohli, James Bruton, Thomas Hallowell, Nile Delso, Anna Roberts, Yuxi Lin, Johannes K. Ehinger, Michael Karlsson, Robert A. Berg, Ryan W. Morgan, Todd J. Kilbaugh

**Affiliations:** 1 Resuscitation Science Center of Emphasis, The Children’s Hospital of Philadelphia, 3401 Civic Center Boulevard, Philadelphia, PA 19104 USA; 2 Department of Anesthesiology and Critical Care Medicine, The Children’s Hospital of Philadelphia, Philadelphia, USA; 3 Department of Cardiology, Pulmonology, and Vascular Medicine, University Hospital Düsseldorf, Medical Faculty of the Heinrich Heine University Düsseldorf, Düsseldorf, Germany; 4 CARID, Cardiovascular Research Institute Düsseldorf, Medical Faculty of the Heinrich-Heine-University, Düsseldorf, Germany; 5 Division of Neurology, The Children’s Hospital of Philadelphia, Philadelphia, USA; 6 Department of Pediatrics, The Children’s Hospital of Philadelphia, Philadelphia, USA; 7 Proteomics Core Facility, The Children’s Hospital of Philadelphia, Philadelphia, USA; 8 Department of Pathology, University of Iowa Carver College of Medicine, Iowa City, IA USA; 9 Mitochondrial Medicine, Department of Clinical Sciences Lund, Lund University, Lund, Sweden; 10 Otorhinolaryngology, Department of Clinical Sciences Lund, Lund University, Lund, Sweden; 11 Otorhinolaryngology, Head and Neck Surgery, Skåne University Hospital, Lund, Sweden; 12 Neurosurgery, Rigshospitalet, Copenhagen, Denmark

**Keywords:** Cardiac arrest, Asphyxia, Dimethyl fumarate, Heart, Brain, Metabolism, Mitochondria, Post-arrest care, Molecular medicine, Paediatric research

## Abstract

Neurological and cardiac injuries are significant contributors to morbidity and mortality following pediatric in-hospital cardiac arrest (IHCA). Preservation of mitochondrial function may be critical for reducing these injuries. Dimethyl fumarate (DMF) has shown potential to enhance mitochondrial content and reduce oxidative damage. To investigate the efficacy of DMF in mitigating mitochondrial injury in a pediatric porcine model of IHCA, toddler-aged piglets were subjected to asphyxia-induced CA, followed by ventricular fibrillation, high-quality cardiopulmonary resuscitation, and random assignment to receive either DMF (30 mg/kg) or placebo for four days. Sham animals underwent similar anesthesia protocols without CA. After four days, tissues were analyzed for mitochondrial markers. In the brain, untreated CA animals exhibited a reduced expression of proteins of the oxidative phosphorylation system (CI, CIV, CV) and decreased mitochondrial respiration (p < 0.001). Despite alterations in mitochondrial content and morphology in the myocardium, as assessed per transmission electron microscopy, mitochondrial function was unchanged. DMF treatment counteracted 25% of the proteomic changes induced by CA in the brain, and preserved mitochondrial structure in the myocardium. DMF demonstrates a potential therapeutic benefit in preserving mitochondrial integrity following asphyxia-induced IHCA. Further investigation is warranted to fully elucidate DMF’s protective mechanisms and optimize its therapeutic application in post-arrest care.

## Introduction

Each year, more than 15,000 children in the United States experience an in-hospital cardiac arrest (IHCA) and receive cardiopulmonary resuscitation (CPR)^1–3^. Of these, 42–52% have an underlying respiratory cause, and one-third will have ventricular fibrillation (VF) at some point during CPR^4–8^. Most children experiencing an IHCA do not survive to hospital discharge, and few survive without significant neurological injury or decline^3,9–12^. A common cause of morbidity and mortality after CA is brain injury, with cardiac dysfunction further contributing significantly^13–16^. Despite this, pharmacological interventions that confer neurological and cardioprotection following CA or acute ischemic stroke are still lacking^17–22^.

CA causes acute hypoxia in multiple organs^23^. The brain and heart are particularly sensitive to the resulting ischemia–reperfusion (IR) injury due to their high metabolic activity^24–27^. While early reperfusion is essential for survival following CA, the resurgence of oxygen during the reperfusion phase causes the formation of excessive reactive oxygen species (ROS). These ROS directly impair mitochondria and initiate a cascade of cell death and pro-inflammatory pathways responsible for IR injury^17,28–34^. Previous work has demonstrated the ability of hemodynamic-directed CPR (HD-CPR) to improve outcomes, in part by preserving mitochondrial bioenergetics^12,35–38^. This indicates that preserving mitochondrial health may be an important convergence point for cell survival. Therefore, we hypothesized that mitochondria-targeted therapeutics may improve mitochondrial metabolism in brain and heart after IHCA and HD-CPR.

**Figure 1:**
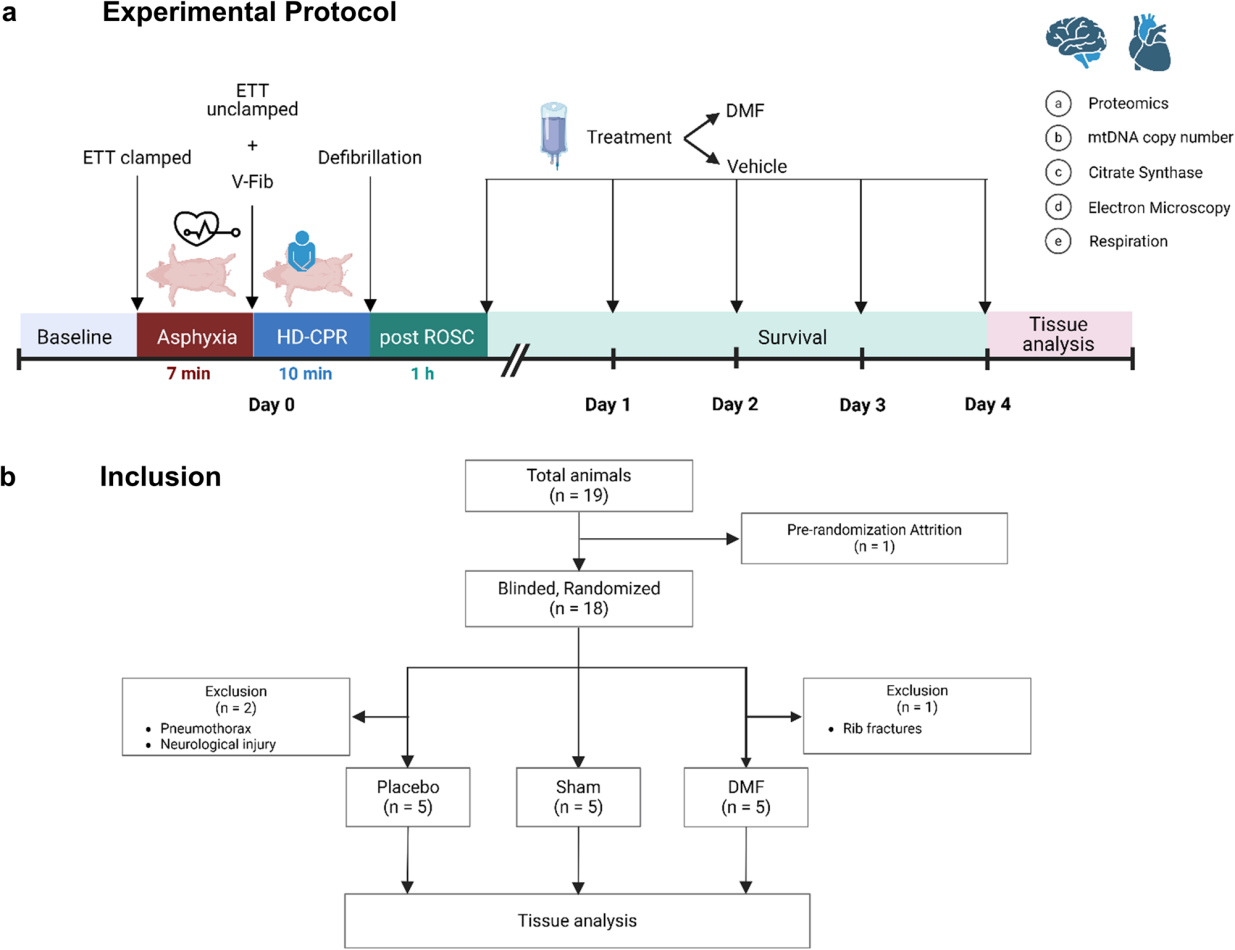
Study design and experimental protocol. (**a**) One-month old piglets were anesthetized, mechanically ventilated and body temperature was maintained between 38–40 °C. Vascular catheters were placed to facilitate hemodynamic assessments, obtain arterial blood gas samples, and measure cardiac output. To simulate asphyxia-induced in-hospital cardiac arrest, piglets underwent a 7-min period of asphyxia by clamping of the endotracheal tube (ETT), followed by induction of ventricular fibrillation (V-Fib) and 10 min of hemodynamic-directed cardiopulmonary resuscitation (HD-CPR) to mimic a clinically relevant insult and administration of high-quality CPR. Vasopressors were giving on an as-needed basis and defibrillation was performed to achieve return of spontaneous circulation (ROSC) beginning ten minutes into CPR and up to every two subsequent minutes as needed. One hour following ROSC, animals were randomized and received either (1) dimethyl fumarate (DMF, 30 mg/kg) or (2) placebo (equivalent volume of the vehicle: 0.9% saline) treatment. Treatments were administered per intravenous infusion through a central venous catheter over a period of 1 h. After administration of placebo or DMF, the animals were weaned off anesthesia, extubated, and returned to their home cage upon clinical stabilization, at an average post-ROSC time of 3 h. Subsequent doses of DMF or placebo treatment were given once daily for four days through the central venous catheter line without additional anesthesia. Sham animals underwent identical anesthesia protocols, venous and arterial line placement and continuous measurements. Four days post-cardiac arrest, the animals were euthanized, and tissues were collected for analysis of molecular markers. The experimental design protocol illustration was created using BioRender.com and Eucalyp from Noun project. (**b**) Of 19 animals entering this study, four animals reached predetermined humane endpoints prior to the study endpoint and were excluded from analysis. A total of five animals per group were consequently included for data analysis.

Dimethyl fumarate (DMF), a derivative of the TCA-cycle intermediate fumaric acid, has anti-inflammatory and antioxidant properties in and beyond the central nervous system^27,39–46^. It is clinically approved as therapy for the treatment of psoriasis and relapsing/relapsing–remitting forms of multiple sclerosis and has been clinically and pre-clinically investigated for treatment of a variety of pathologies, including amyotrophic lateral sclerosis, glioblastoma multiforme, Parkinson’s disease and multiple forms of cancer^47,48^. Only recently has it gained attention as potential treatment for cardiovascular disease due to its anti-inflammatory and antioxidant properties, with a particular emphasis on heart-related conditions^27,49–51^. Moreover, it has shown to increase mitochondrial content and function through activation of the nuclear respiratory factor 1 and 2 (NRF1, NRF2) pathway, while also mitigating oxidative damage through the upregulation of antioxidant response elements^52–56^. Because previous work has demonstrated that mitochondrial function and content continue to be dysregulated despite successful resuscitation after asphyxia-induced IHCA, and that preservation of mitochondrial function and dynamics correlates with good neurologic outcome after asphyxia-induced IHCA and CPR^36,37,57,58^, we hypothesized that DMF would increase mitochondrial function in brain and heart after asphyxia-induced CA and HD-CPR. To test this hypothesis, we performed a randomized preclinical trial with DMF in a pediatric porcine model of asphyxia-induced IHCA^59^ and evaluated its treatment efficacy based on changes in mitochondrial content and function, oxidative damage, and the cerebral proteome as outcome measures. The complete study design is illustrated in Fig. 1a.

## Results

### Relevant physiological pre-randomization characteristics did not differ between groups

Of the 19 animals entering this study, four animals reached pre-determined humane endpoints prior to the study endpoint and were excluded from analysis: (1) one animal did not achieve return of spontaneous circulation (ROSC), (2) two animals of the placebo group achieved ROSC but suffered from pneumothorax or severe neurological injury requiring humane euthanasia and (3) one animal of the DMF group achieved ROSC but suffered from rib fractures shortly after ROSC (Fig. 1b). For all animals reaching the experimental endpoint (n = 5 per group), i.e. survival until after the last treatment dose of placebo or DMF on day four following successful resuscitation from CA, the results of hemodynamic and venous blood gas parameters of baseline, asphyxia, CPR, and ROSC periods are shown in Supplementary Table S4. Relevant systemic physiologic and pre-randomization characteristics did not differ between any of the groups at baseline. During asphyxia and CPR, there were no differences between the two treatment groups (placebo and DMF). CPR duration, number of required vasopressor doses, and the number of defibrillation attempts were consistent between the two groups (Supplementary Table S5). Within one hour after ROSC and before administration of DMF or placebo, blood gases and hemodynamics had normalized and were similar between groups, with the exception of diastolic blood pressure, which was significantly higher in the DMF group compared to sham and placebo groups (Supplementary Table S4).

### DMF treatment prevented long-term proteomic changes in the brain

**Figure 2:**
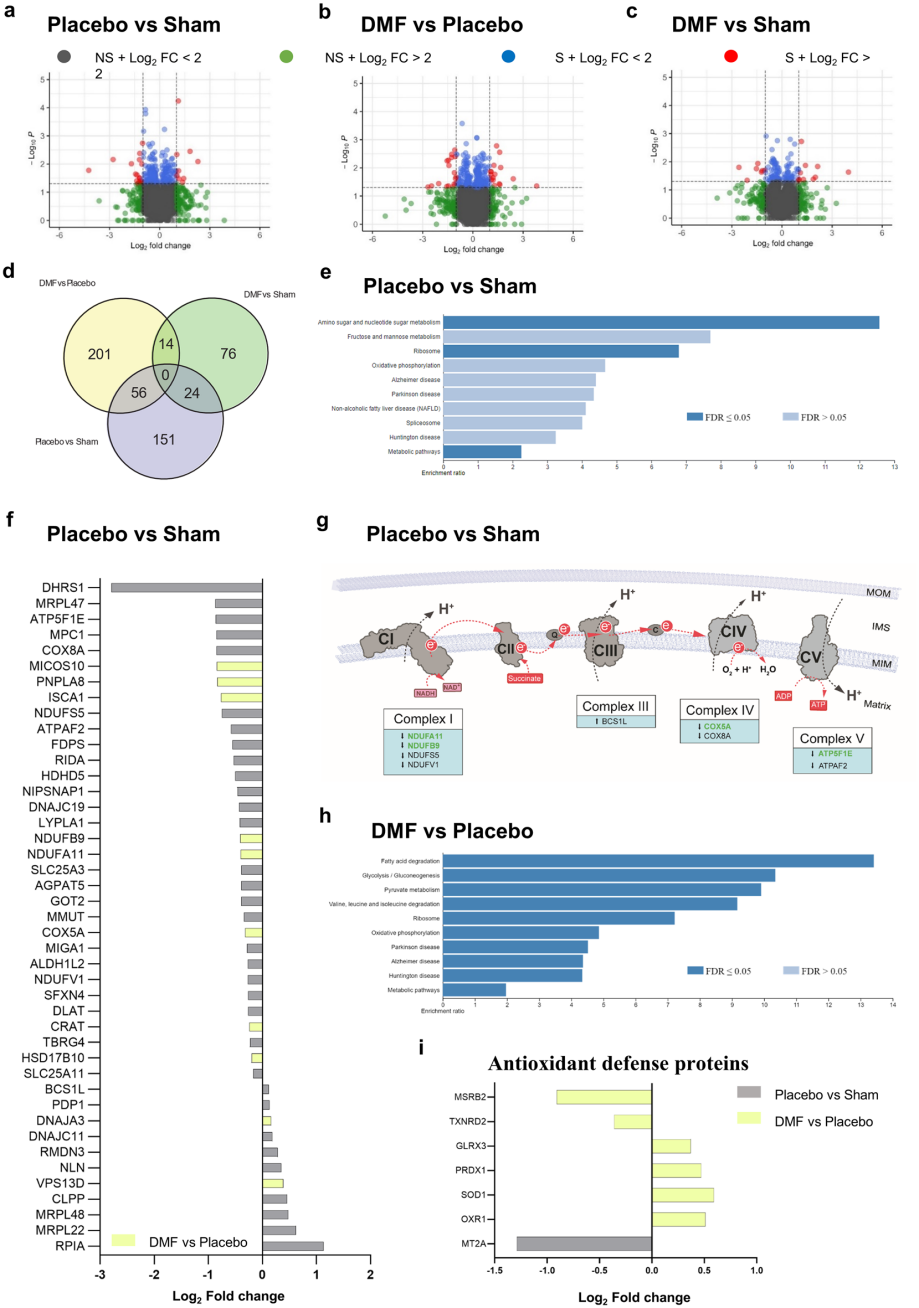
Cerebral proteomic profile. Protein expression was measured in cortex of sham (Sham) and cardiac arrest animals who received either placebo (Placebo) or dimethyl fumarate (DMF) treatment. A *t*-test was subsequently employed to identify differentially expressed proteins between groups using p value < 0.05 as significant threshold. Volcano plots of the differential protein expression of (**a**) Placebo and Shams, (**b**) DMF and Placebo and (**c**) DMF and Shams. Non-significant protein changes below and above a Log_2_ Fold Change (FC) of 2 are indicated by gray (NS + Log_2_ FC < 2) and green (NS + Log_2_ FC > 2) data points, whereas blue (S + Log_2_ FC < 2) and red (S + Log_2_ FC > 2) data points indicate significant protein changes below and above a Log_2_ FC of 2, respectively. (**d**) Venn diagram showing the number of all overlapping protein changes between groups. (**e**) Pathway enrichment analysis performed using KEGG of downregulated proteins of placebo animals compared to Shams. (**f**) Log_2_ FC change of up- and downregulated mitochondrial proteins in placebo animals compared to shams with changes of DMF compared to placebo animals highlighted in yellow. (**g**) Illustration of proteomic changes of the oxidative phosphorylation (OXPHOS) system in placebo compared to Shams with proteins that were rescued by DMF highlighted in green. (**h**) Pathway enrichment analysis of upregulated proteins of DMF compared to placebo animals. (**i**) Log_2_ FC change of up- and downregulated proteins involved in cellular antioxidant defense of placebo animals compared to Shams and DMF compared to placebo animals. *FDR* false discovery rate, *NS* not significant, *S* significant. n = 5.

Proteomic analysis of the cortex was performed at the experimental endpoint, i.e. after the last treatment dose of placebo or DMF on day four following successful resuscitation from asphyxia-induced IHCA. It revealed a significantly altered proteomic profile (Fig. 2, Supplementary Table S6). Of 231 proteins significantly altered in placebo compared to sham animals, 139 (60%) were downregulated and 92 (40%) were upregulated (Fig. 2a,d, Supplementary Table S6). Pathway-enrichment analysis revealed significant changes in nucleotide metabolism and metabolic and ribosomal pathways following asphyxia-induced IHCA (all FDR ≤ 0.05) (Fig. 2e, Supplementary Table S7). Twenty percent of all proteins changed by asphyxia-induced IHCA were mitochondrial, and 86% of these were downregulated (Fig. 2f, Supplementary Table S6). Downregulated mitochondrial proteins included subunits and assembly factors of mitochondrial complex I, IV and V, metals and cofactors, proteins involved in carbohydrate and lipid metabolism and mitochondrial translation, protein homoeostasis and dynamics (Fig. 2f,g, Supplementary Table S6), some of which are associated with common neurodegenerative diseases. Only 11 mitochondrial proteins were upregulated following asphyxia-induced IHCA, including oxidative phosphorylation (OXPHOS) complex assembly factors, proteins of carbohydrate metabolism, mitochondrial ribosomal proteins and proteins involved in mitochondrial protein homeostasis and dynamics (Fig. 2f, Supplementary Table S6). We also evaluated changes in proteins involved in cellular antioxidant defense by comparing the list of significantly altered proteins in our data set with proteins previously reported to be altered through the NRF1, NRF2 pathway^60–63^ and found the antioxidant protein MT2A to be reduced following asphyxia-induced IHCA (Fig. 2i, Supplementary Table S6).

DMF treatment prevented 25% of the proteomic changes induced by asphyxia-induced IHCA in the cortex of the brain (Fig. 2, Supplementary Table S6). Of these, 20% were localized to the mitochondrion, including subunits of complex I, IV and V, metals and cofactors, proteins involved in mitochondrial structure, as well as few proteins involved in mtRNA and substrate metabolism (Fig. 2f, Supplementary Table S6). Four key antioxidant defense proteins were upregulated in response to DMF (OXR1, SOD1, PRDX1 and GLRX3) (Fig. 2i, Supplementary Table S6). Pathway-enrichment analyses showed an upregulation of proteins associated with the OXPHOS system, fatty acid degradation, glycolysis/gluconeogenesis, pyruvate metabolism, metabolic pathways, ribosomal pathways and valine, leucine and isoleucine degradation by DMF compared to placebo-treated CA animals (FDR ≤ 0.05: Fig. 2h, Supplementary Table S7). DMF treatment furthermore counteracted the downregulation of proteins associated with classical neurodegenerative diseases (Fig. 2h, Supplementary Table S7). Proteins involved in the NRF1/NRF2 pathway, a key pathway known to play a role in IR injury and targeted by DMF^52–56,64^, were unchanged by asphyxia-induced IHCA. A full list of proteomic changes between the different treatments groups is shown in Supplementary Table S6.

### Myocardial mitochondrial morphology was preserved by DMF treatment

**Figure 3:**
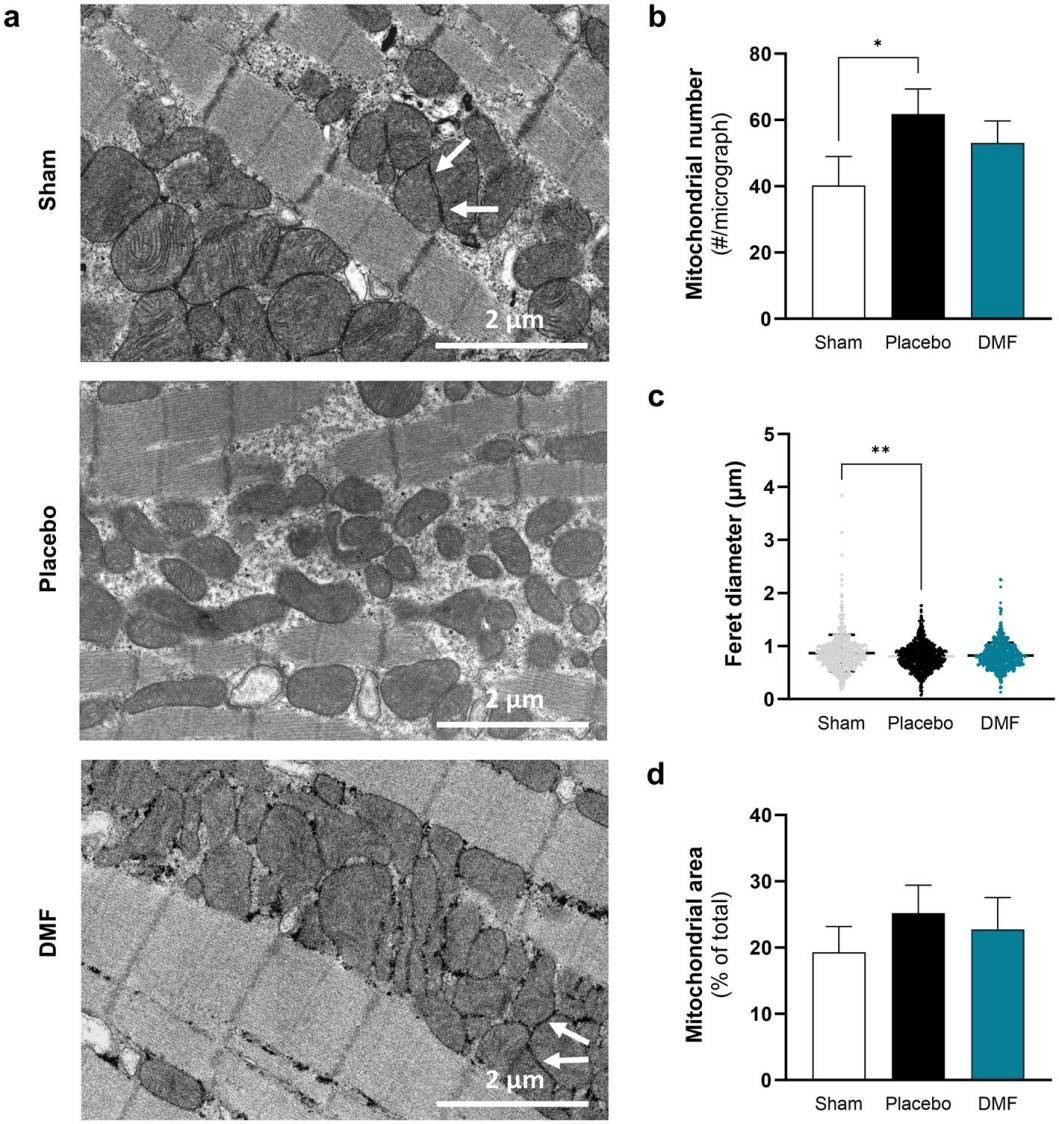
Myocardial transmission electron microscopy. Mitochondrial content and morphology of the myocardium was analyzed by transmission electron microscopy. (**a**) Representative images from sham, (top), placebo (middle) and DMF (bottom) -treated animals are shown. Image analysis allowed the comparison of (**b**) mitochondrial content (# per micrograph), (**c**) mitochondrial elongation (feret diameter, µm) and (**d**) mitochondrial area (% of total area) between groups. White arrows indicate inter-mitochondrial junctions between adjacent mitochondria. Normally distributed data were statistically analyzed using ordinary one-way ANOVA (homogenous variances) or Brown–Forsythe and Welch ANOVA (non-homogenous variances). For comparisons of non-normally distributed data, Kruskal–Wallis test was applied. Every group was compared to every other group. *p < 0.05 and **p < 0.01. Data are presented as mean ± SD. n = 5.

Four days after asphyxia-induced IHCA, there was no difference in Citrate Synthase (CS) activity between treatment groups in brain and myocardium (Supplementary Fig. S1). Relative mtDNA copy number was consistently lower following asphyxia-induced IHCA in both tissues, yet not significantly (Supplementary Fig. S2). We additionally measured CS activity and relative mtDNA copy number in kidney and soleus muscle in order to further strengthen potential findings and investigate whether the large within group variation in relative mtDNA copy number is experimentally related or due to an inherently different mtDNA copy number between animals (Supplementary Fig. S1 and S2). We observed no significant differences in relative mtDNA copy number in the kidney or soleus muscle, nor in CS activity in the kidney. Interestingly, the CS activity in soleus muscle was significantly reduced by asphyxia-induced IHCA (− 48%, p < 0.05 for Placebo vs. Sham), which was prevented by DMF treatment (Supplementary Fig. S1). Ultrastructural analysis by transmission electron microscopy (TEM), the gold standard for evaluation of mitochondrial content and structure, revealed an increase in mitochondrial number in the left ventricle (LV) (Fig. 3a, representative images) following asphyxia-induced IHCA (p = 0.02 for Placebo vs. Sham) (Fig. 3b). Despite the higher number, mitochondrial elongation was reduced (Feret’s diameter) after asphyxia-induced IHCA (Fig. 3b,c). While the total percentage area of myocardium occupied by mitochondria remained constant (Fig. 3d), these morphological changes contributed to fragmentation of the mitochondrial network, indicating an imbalance in mitochondrial dynamics with a shift towards mitochondrial fission. DMF preserved the mitochondrial network compared with Placebo, maintaining inter-mitochondrial junctions between adjacent mitochondria and structural integrity of the myocardium (Fig. 3a)^65^. DMF also normalized mitochondrial content and shape, but had no effect on mitochondrial size distribution (Fig. 3, Supplementary Fig. S3).

### Mitochondrial function remains impaired despite DMF treatment

**Figure 4:**
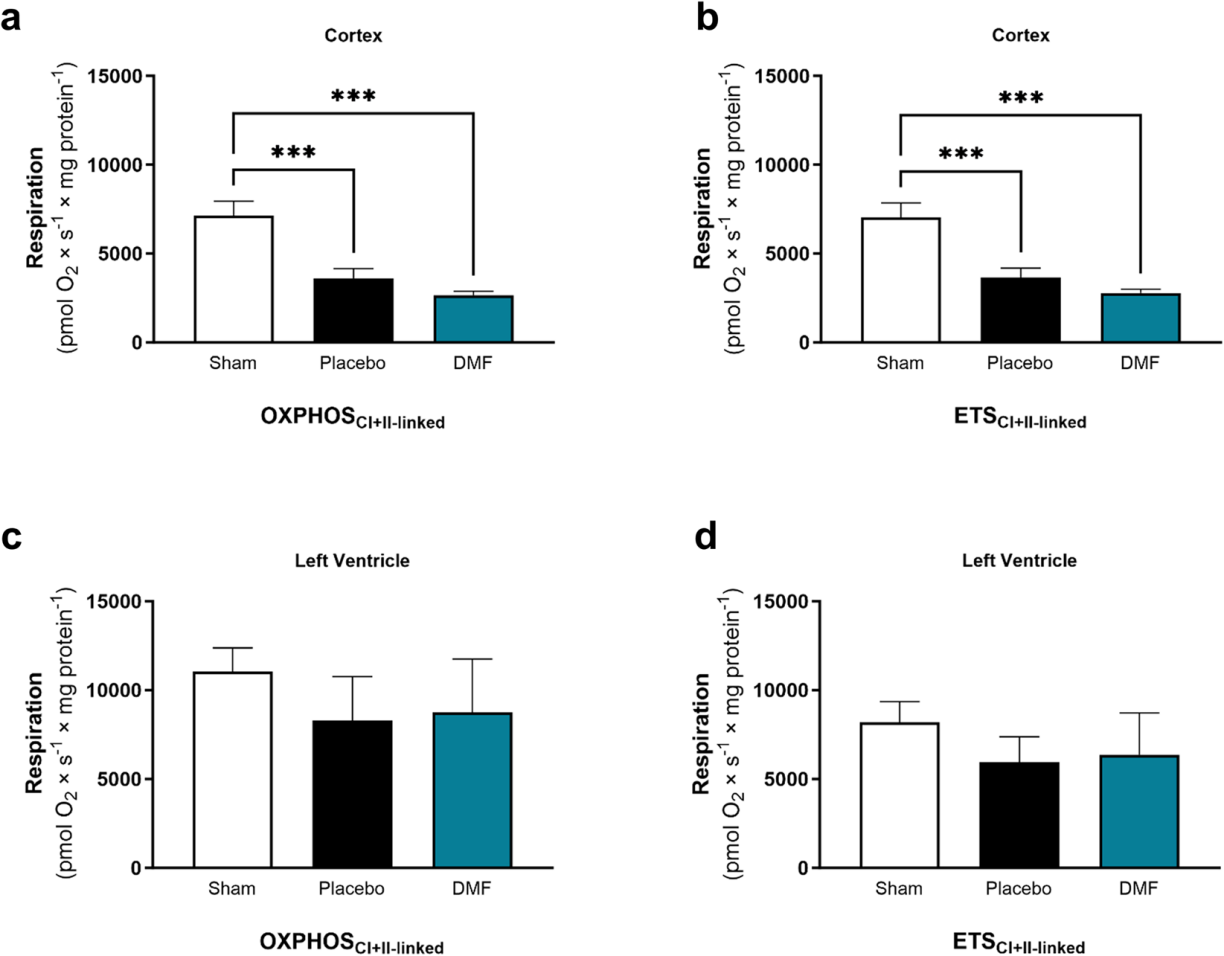
Mitochondrial function. Mitochondrial respiration of isolated mitochondria from brain (cortex, 25 µg/mL) and heart (left ventricle, 16 µg/mL) was assessed in sham animals (Sham) and cardiac arrest animals who received either placebo (Placebo) or dimethyl fumarate (DMF) treatment. In-depth characterization of mitochondrial function was performed using high-resolution respirometry and a Substrate-Uncoupler-Inhibitor Titration protocol. (**a**,**c**) Maximal complex I + II-linked oxidative phosphorylation capacity (OXPHOS) as well as (**b**,**d**) electron transport system (ETS) capacity is shown. Mitochondrial respiration is expressed as pmol O_2_ × s^−1^ × mg protein^−1^. Normally distributed data were statistically analyzed using ordinary one-way ANOVA (homogenous variances) or Brown–Forsythe and Welch ANOVA (non-homogenous variances). For comparisons of non-normally distributed data, Kruskal–Wallis test was applied. Every group was compared to every other group. Data are presented as mean ± SD. n = 5. ***p < 0.001. *CI + II* convergent complex I and II. n = 5.

Brain mitochondria showed reduced respiration following asphyxia-induced IHCA, regardless of treatment. Assessment of mitochondrial respiration revealed significantly decreased OXPHOS and Electron Transport System (ETS) capacity in cortex and hippocampus of placebo-treated CA animals compared to Shams (Fig. 4a,b, Supplementary Fig. S4). The same trends were observed in homogenates of cortex and hippocampus (Supplementary Figs. S5, S6), suggesting these results were not simply due to analysis of a subpopulation of isolated mitochondria^66^. In contrast to brain, myocardial mitochondria showed no significant difference between placebo animals and Shams four days post-CA (Fig. 4c,d, Supplementary Fig. S4). Overall, DMF had no appreciable effect on mitochondrial respiration (Fig. 4, Supplementary Figs. S4–S6). There were no significant changes in oxidative damage markers (protein carbonyls and 3-nitrotyrosine) between groups in brain and heart (Supplementary Fig. S7).

## Discussion

Cardiac arrest causes systemic IR injury, and mitochondria are critical determinants of the resulting pathology^23^. However, little is known on mitochondrial alterations in the brain and heart past the acute phase (> 48 h). In this study, we evaluated the effects of CA on mitochondrial health in multiple organ systems four days after CA using a pediatric porcine model of asphyxia-induced IHCA. To our knowledge, this is the first study to provide a comprehensive mitochondrial profile of both brain and heart more than 48 h after successful resuscitation from CA. Our results demonstrate that mitochondrial content and function are impaired for at least four days following asphyxia-induced IHCA in skeletal muscle and brain, respectively. In contrast, myocardial mitochondria, previously reported to be impaired up to 24 h post-CA^14,37,58^, had partially recovered by four days after asphyxia-induced IHCA. We observed no difference in mitochondrial function despite persistent changes in mitochondrial content and ultrastructure, as assessed per TEM. Taken together, the results suggest increased resilience of myocardial mitochondria to IR injury relative to other organs. DMF treatment, which has recently gained attention as potential treatment for cardiovascular disease due to its anti-inflammatory and antioxidant properties but whose effects on multiple mitochondrial markers have not been investigated in the context of CA before, partially ameliorated IHCA-associated mitochondrial injuries. This included the prevention of the proteomic changes in the brain and restoration of mitochondrial morphology and content in the myocardium and soleus muscle, respectively.

The brain is particularly sensitive to IR injury, presumably due to its high metabolic rate and relatively low antioxidant defenses^24,25^. To our knowledge, this is the first study to provide a comprehensive mitochondrial profile of the brain four days after successful resuscitation in a large animal model of asphyxia-induced IHCA. Advancements in high-quality CPR and post-arrest care have shown great promise in animals and humans and represent an avenue for continued innovation in this field^12,35–37,57,67–69^. Our previous work has demonstrated that HD-CPR improved neurological outcomes in a pediatric porcine model of asphyxia-induced IHCA, in part by preserving mitochondrial bioenergetics^12,35–38^. Despite excellent HD-CPR and provision of continuous organ perfusion after onset of CA, here we show that mitochondrial protein expression continued to be significantly altered in the brain four days after asphyxia-induced IHCA. Key changes included the downregulation of mitochondrial OXPHOS complexes and assembly factors, as well as proteins involved in mitochondrial translation, metabolism, dynamics, and antioxidant response. These proteomic changes may underlie the chronic impairment in mitochondrial bioenergetics in the brain following asphyxia-induced IHCA. While brain injury following CA is the primary cause, cardiac dysfunction also contributes significantly to morbidity and mortality following CA^3,14,16^. In this study, we observed a disturbance of mitochondrial content and structure in the heart, while mitochondrial function remained intact. Our findings align with previous research, indicating that cardiac and cerebral mitochondrial dysfunction after CA follow a different time-course and pattern, and that the brain shows a higher sensitivity to damage caused by CA^14,16^. Myocardial dysfunction due to ischemia can be transient and potentially reversible depending on the duration of ischemia and time to ROSC. This recovery response of the so called “stunned myocardium” has been described for situations where ischemia is resolved before necrotic and apoptotic events are initiated, such as during VF-induced CA. And yet, the exact mechanisms by which the myocardium counteracts the transient period of ischemia are still not completely understood^17,70–73^. Interestingly, despite the absence of a functional deficit in the myocardium, TEM ultrastructural analysis revealed an increased number of mitochondria and fragmentation of the mitochondrial network (Fig. 3). An increased mitochondrial fragmentation is indicative of an imbalance in mitochondrial dynamics with a shift towards mitochondrial fission. An increasing body of evidence suggest impaired mitochondrial dynamics in the heart to be associated with the development of cardiovascular disease^74,75^. The normalization of the mitochondrial networked, as observed in this study, could therefore potentially be beneficial. However, more research is needed addressing this scientific questions. The data presented here clearly demonstrate that the distinct metabolic profiles of brain and heart may require tailored intervention strategies specifically designed for each organ.

Prior studies have shown that DMF treatment can improve mitochondrial bioenergetics and reduce oxidative damage in in vitro and in vivo in models of Parkinson’s disease, multiple sclerosis and Friedrich’s Ataxia^52,53,56,76^. Because these diseases share common underlying neuropathological mechanisms with IR injury, we hypothesized that DMF may show beneficial effects in heart and brain following asphyxia-induced IHCA as well. Indeed, DMF treatment prevented pathogenic changes in mitochondrial proteins central to OXPHOS, fatty acid and substrate metabolism, and mitochondrial dynamics, while boosting antioxidant defenses and proteins associated with neurodegenerative disease. Surprisingly, DMF failed to rescue the persistent decline in mitochondrial function following asphyxida-induced IHCA in the brain. In the heart, DMF prevented fragmentation of the mitochondrial network and maintained myocardial ultrastructure, the significance of which remains to be elucidated in future studies. We chose to evaluate the effect of DMF on mitochondrial health in a pediatric porcine model of asphyxida-induced IHCA because respiratory deterioration accounts for 42–52% of all pediatric cases of IHCA^4–9,55,77^. The induction of VF allowed us not only to provide and standardize high-quality CPR and post-arrest care to enhance the probability to detect changes in response to DMF treatment but also holds significant relevance for the clinical setting with almost one-third of all IHCA cases presenting with VF during CPR^36,78^. The CA period of 10 min simulates a realistic clinical scenario^78^. However, previous studies have shown that the time of ischemia and time to ROSC can affect IR damage, resuscitation, and recovery from CA. Furthermore, current out-of-hospital cardiac arrest (OHCA) treatment strategies instead focus primarily on improving bystander CPR and basic life support during the pre-hospital phase and likely are characterized by more pronounced IR injury^3,79,80^. Therefore, it remains unknown whether DMF would induce comparable effects on mitochondrial metabolism in the brain and heart in OHCA models and IHCA models with different etiology. Although both IHCA and OHCA entail IR injury, the resulting pathology is influenced by the cause and duration of ischemia, time to ROSC, comorbidities and other factors that vary between IHCA and OHCA, as well as between pediatric and adult CA^3,79,80^.

Patients who survive critical illness not only experience neurological decline, but also muscular atrophy, nerve damage, and deconditioning, known as intensive care unit-acquired weakness (ICA-AW)^81–84^. ICA-AW is associated with increased morbidity and mortality. Most of the cellular events underlying this condition have been linked to mitochondrial dysfunction^85–87^. The decline in mitochondrial health correlates with poor prognosis following critical illness^88,89^. DMF prevented loss of mitochondria in the soleus muscle following IHCA. Whether the mitochondrial protection by DMF would be sufficient to counteract ventilator-induced diaphragmatic dysfunction or skeletal muscle atrophy associated with ICU-AW warrants further investigation.

In this study, we evaluated mitochondrial content by CS activity, relative mtDNA copy number and TEM. We hypothesized that a correlation between these markers would further strengthen our data. To our surprise, the above selected biochemical markers of mitochondrial content showed little correlation in heart and soleus muscle (Fig. 3, Supplementary Figs. S1, S2). These results highlight that selecting the right marker of mitochondrial content is therefore crucial. Mitochondrial content is not only important for normalization of data on mitochondrial function, but it also serves as a prevalent marker of bioenergetic health on its own^90–92^. To date, the gold standard for evaluation of mitochondrial content is still TEM^90^. TEM, however, is time consuming, costly and requires specialized equipment and expertise not available at every institution^90,93^. Different biochemical markers of mitochondrial content have therefore been proposed as surrogate markers. The most commonly used biochemical markers of mitochondrial content currently are CS activity and -content, and relative mtDNA copy number. However, studies have shown that these different biochemical markers often do not correlate with each other^90,91,94–97^. Few studies have used and compared more than one marker of mitochondrial content. The only comprehensive comparison of mitochondrial markers was done by Larsen et al.^90^, who studied the correlation of more than ten different biochemical markers with analysis by TEM. His study showed the strongest correlation between mitochondrial content as determined by TEM analysis and the biochemical markers cardiolipin content and CS activity in skeletal muscle of healthy human subjects. Furthermore, studies indicate that relative mtDNA copy number might be a poor biochemical marker of mitochondrial content^90,91^. This is partially due to the large variation in the copy number of the mitochondrial genome between individuals^90^, which was also demonstrated by the data presented in this manuscript. While relative mtDNA copy number showed no statistical difference between groups, all organs demonstrated the same large within group variation (Supplementary Fig. S2). Correlation analysis of the relative mtDNA copy number of heart, kidney and soleus muscle of each animal with the relative mtDNA copy number of the brain of the same animal revealed that the large variation is due to an inherently different copy number between animals. Despite the comprehensive study by Larsen et al.^90^, little is known about basic behaviour and correlation of these biochemical markers in other tissues, such as brain, kidney, liver, and adipose tissue^91^. Complexity is added by the fact that any of these markers can change as a result of the disease, age group, and tissue that is studied, and therefore would not be suitable for assessment of mitochondrial quantity and normalization of mitochondrial function^98^. Further work is required to identify the best marker of mitochondrial content in different organs, species, and diseases to advance the research in this area.

Our study had certain limitations: First, statistical power may have been limited by the relatively small sample size. In addition, the dose of DMF selected for this study was based on previously published in vivo rodent data^52^. No further dose-titration and optimization was performed. At last, DMF is known to possess anti-inflammatory properties. Effects of DMF on the inflammatory status of the brain and heart was outside the scope of the present study. It therefore remains unknown if DMF potentially would induce beneficial anti-inflammatory effects in the settings of pediatric, asphyxia-induced CA.

In conclusion, we establish that mitochondrial injury persists in the brain, heart and skeletal muscle at four days following asphyxia-induced IHCA, and that DMF can partially mitigate the mitochondrial injury. No increase in oxidative damage markers was observed in asphyxia-induced CA animals. Although DMF was able to ameliorate some of the mitochondrial alterations in brain and heart following CA and increased the expression of antioxidant proteins, future studies are required to better assess DMF as a mitochondrial therapeutic for CA and ICU-AW.

## Materials and methods

### Ethical declaration

The study was approved by the Institutional Animal Care and Use Committee at the Children’s Hospital of Philadelphia (study number: 1236, study title: Advanced Therapies in Pediatric Cardiac Arrest, approval date: 9/25/2019) and performed in accordance with the National Institutes of Health (NIH) Guide for the Care and Use of Laboratory Animals and ARRIVE guidelines. Our study was designed to simulate IHCA following respiratory deterioration in a young child, as respiratory deterioration is a frequent precursor of pediatric IHCA^9,77^.

### Animal preparation

One-month old Yorkshire piglets (*Sus scrofa domestica*) representing toddler age were selected due to the similarities to humans in neurodevelopment, gross anatomical and cerebral physiological features, and chest compression characteristics^36^. Piglets were anesthetized and mechanically ventilated at room air and 1–2.5% isoflurane to maintain anesthesia (Modulus SE; Datex Ohmeda, Madison, WI). They were continuously ventilated at a tidal volume of 10 mL/kg, positive end-expiratory pressure of 6 cm H_2_O and respiratory rate was titrated to maintain an end-tidal CO_2_ of 38–42 mmHg (NICO; Novametrix Medical Systems Inc., Wallingford, CT). Under ultrasound guidance, vascular catheters were placed to facilitate hemodynamic assessments, obtain arterial blood gas samples, and measure cardiac output. A central venous catheter (CVC) was additionally placed for blood collection and drug administration purposes. To prevent catheter clotting, unfractionated heparin (200U/kg) was administered. Body temperature was maintained between 38–40 °C throughout the experimental procedures using a heating blanked (TP700, Stryker Medical). Hemodynamic parameters, pulse oximetry, end-tidal carbon dioxide (ETCO_2_) and temperature were recorded using PowerLab (ADInstruments)^36,78^.

### Experimental protocol

We used a previously published pediatric porcine model of IHCA^59^, which simulates an asphyxial event followed by VF. Respiratory deterioration accounts for 42–52% of all pediatric cases of IHCA^4–9,77^. Furthermore, VF is a frequent event during IHCA, accounting for almost one-third of all IHCA cases, and therefore holds significant relevance for CA in children in the clinical setting. Following 5 min of stable baseline readings and administration of ketamine (3 mg/kg/h, IV), dexmedetomidine (5 μg/kg/h, IV) and buprenorphine (0.02 mg/kg, IM), 7 min of asphyxia were induced by clamping of the tracheal tube and confirmed by the absence of exhaled CO_2_. The 7-min asphyxia period was selected with the intent to cause severe arterial hypoxemia and hypercapnia. At the end of the asphyxia period, VF was induced via transthoracic intracardiac needles, confirmed by electrocardiogram, and HD-CPR was immediately initiated. At the same time, the tracheal tube was unclamped and mechanical ventilation was restarted at a FiO_2_ of 100%. VF was maintained for 10 min to allow for a standardized period of high-quality CPR in both CA groups. Furthermore, the CA period of 10 min simulates a realistic clinical scenario. HD-CPR has previously shown to increase short-term survival rates in comparison to standard, depth-guided CPR^36^. The rate and depth of chest compression was continuously titrated in each animal to achieve a target systolic blood pressure (SBP) of 90 mmHg. We selected 90 mmHg of SBP based on previously reported SBP values observed during successful CPR in children with IHCA^99^ and porcine models of CA^100–102^. Vasopressors (epinephrine [0.02 mg/kg] and vasopressin [0.4 U/kg]) were giving when needed to maintain a coronary perfusion pressure (CoPP) above 20 mmHg^57,59^. The first defibrillation attempt was made by administering 50 J (5 J/kg, Zoll R Series, Zoll Medical Corporation) to achieve ROSC. This shock dose has previously shown to be safe and effective in piglets^101^. In animals not achieving ROSC, HD-CPR was continued for up to a maximum of 20 min, with defibrillation every 2 min. If no ROSC was achieved in this period, the animals were humanely euthanized. In animals with ROSC, a standardized post-arrest care protocol was performed, in which mechanical ventilation was provided to maintain an ETCO_2_ of 38–42 mmHg, SpO_2_ 94–99%, and isoflurane was continuously titrated to keep the animals anesthetized, as evaluated per lack of a toe-pinch reflex response. MAP was maintained at > 90 mmHg by use of vasopressors. The porcine model of asphyxia-induced IHCA is described in more detail in previous work^36,37^.

One hour following ROSC, CA animals were randomized into (1) DMF or (2) Placebo group. DMF treatment (30 mg/kg) or vehicle (equivalent volume) was administered per intravenous infusion through the cephalic vein via CVC line over a duration of one hour. The concentration of the DMF infusion used was 1.6 mg/mL, accounting for an infusion volume of 180 mL of DMF or vehicle (0.9% saline). Throughout the infusion period, animals were kept anesthetized and mechanically ventilated as described above. After administration of placebo treatment or DMF, the animals were weaned off anesthesia, extubated, and returned to their home cage upon clinical stabilization, at an average post-ROSC time of three hours. Subsequent doses of DMF or placebo treatment were given once daily for four days through the CVC line without additional anethesia. The infusion treatment was concealed for the infusion period and could not be determined by the animal personnel. Sham animals underwent identical anesthesia protocols, instrumentation and measurements as CA animals. All personnel handling the animals and downstream analysis were blinded to group assignments, except for one team member who randomized the assignments. The complete study design is illustrated in Fig. 1a.

### Sample acquisition

Following the last treatment dose on day four post-CA, the animals were reanesthesized with ketamine (~ 20 mg/kg, IV), intubated and maintained on inhalant anesthetics (5% isoflurane). A craniotomy was performed to immediately access the brain and prevent cerebral ischemia. The piglets were then humanely euthanized with potassium chloride (~ 2 mEq/kg, IV). Brain (cortex and hippocampus), myocardium (left ventricle and right ventricle), kidney and soleus muscle were simultaneously and rapidly collected. Tissues were preserved in (1) isolation buffer for high-resolution respirometry of fresh tissue^59^, (2) paraformaldehyde-glutaraldehyde solution for TEM or (3) snap frozen on dry ice for further biomarker analysis. An overview of the molecular analyses performed in this study is shown in the Supplementary Table S1.

### Proteomics

Snap frozen cortex was added to 5% sodium dodecyl sulfate/50 mM Triethylammonium bicarbonate buffer containing protease inhibitors (Roche cOmplete) and benzonase (Sigma). Samples were homogenized using 1.4 mm ceramic beads (Omni Scientific) on a Fisher Bead Mill 24 for 20 s at 4.85 m/s, then centrifuged at 15k rcf for 10 min. 200 µg of each sample was digested for S-trap (Protifi) per manufacturer’s protocol. Trypsin (Promega)/LysC (Wako) was added at a ratio of 1:10 and digested for 2 h at 37 °C. Peptides were eluted in 80 μL of 50 mM TEAB followed by 80 μL of 0.1% trifluoroacetic acid (TFA) (Pierce) in water and 80 μL of 50/50 acetonitrile/water (Fisher) in 0.1% TFA. Eluates were combined and desalted using Phoenix peptide cleanup kit (PreOmics) per manufacturer’s protocol, dried by vacuum centrifugation and reconstituted in 0.1% TFA containing indexed retention time (iRT) peptides (Biognosys, Schlieren, Switzerland). All samples were processed identically and exhibited no observable difference in overall protein yield or signal abundance. Following standardization, 2 μg protein of each sample was subjected to mass spectrometry analysis. Samples were analyzed on a QExactive HF mass spectrometer (Thermofisher Scientific San Jose, CA) coupled with an Ultimate 3000 nano UPLC system and an EasySpray source. Data were collected using data independent acquisition (DIA). Tryptic digests were spiked with iRT standards (Biognosys) and separated by reverse phase Reversed-Phase High Performance Liquid Chromatography (RP-HPLC) on a nanocapillary column, 75 μm id × 50 cm 2 μm PepMap RSLC C18 column at 50 °C. Mobile phase A consisted of 0.1% formic acid and mobile phase B of 0.1% formic acid/acetonitrile. Peptides were eluted into the mass spectrometer at 210 nL/min with each RP-HPLC run comprising a 125-min gradient from 1 to 5% B in 15 min, 5–45% B in 140 min. The raw files for DIA were collected using the following settings: one full mass spectrometer scan at 120,000 resolution and a scan range of 300–1650 m/z with an AGC target of 3e6 and a maximum inject time of 60 ms. This was followed by 22 DIA isolation windows with varying sizes at 30,000 resolution, an Automatic Gain Control (AGG) target of 3e6, injection times set to auto. The default charge state was 4, the first mass was fixed at 200 m/z and the normalized collision energy for each window was stepped at 25.5, 27 and 30. The suitability of Q Exactive HF instrument was monitored using QuiC software (Biognosys, Schlieren, Switzerland) for the analysis of the spiked-in iRT peptides. Meanwhile, as a measure for quality control, we injected standard *E*. *coli* protein digest in between samples (one injection after every 6 biological samples) and collected the data in the Data Dependent Acquisition (DDA) mode. The collected DDA data were analyzed in MaxQuant (34) and the output was subsequently visualized using the PTXQC (35) package to track the quality of the instrumentation. The raw files for DIA were processed with Spectronaut^103^ version 15 in DirectDIA mode using reference *Sus scrofa* proteome from the protein database UniProt (49,866 proteins; SwissProt and TrEMBL). Perseus (1.6.14.0) was employed for data processing and statistical analysis using the MS2 intensity values generated by Spectronaut^104^. The data were log_2_ transformed and normalized by subtracting the median for each sample. The proteins with less than 3 valid values in at least one group were filtered out. Student’s *t*-test identified differentially expressed proteins between groups. Proteins with p < 0.05 were subjected to pathway-enrichment analysis (KEGG), mitochondrial proteins were identified using MitoCarta 3.0 and proteins of the Nuclear erythroid-related factor 1/Nuclear erythroid-related factor 2 (NRF1/NRF2) pathway were identified based on the literature^60–63^.

### Mitochondrial function

Mitochondrial function was assessed by means of high-resolution respirometry; see Supplementary Table S2 for details on the Substrate–Uncoupler–Inhibitor Titration (SUIT) protocol. Homogenates of brain tissue and isolated mitochondria of both LV and right ventricle (RV) were prepared as described by Jang et al.^105^. Non-synaptic brain mitochondria were isolated from the cortex and hippocampus as previously described^106,107^. After preparation of the mitochondrial isolates, their respective protein concentration was determined as surrogate for mitochondrial number, and respiration was subsequently assessed at 25 µg/mL and 16 µg/mL of cerebral and cardiac mitochondrial protein, respectively. Respiratory values are expressed as pmol O_2_ × s^−1^ × mg protein^−1^. Mitochondrial respiration of brain homogenates was evaluated at 1 mg/mL wet tissue per chamber, normalized for CS activity as surrogate marker of mitochondrial content and expressed as pmol O_2_ × s^−1^ × CS activity^−1^. Respirometry samples of brain homogenates were subsequently stored at − 80 °C for CS activity and mtDNA copy number quantification.

### Citrate synthase activity

CS activity was independently used as a surrogate marker of mitochondrial content and additionally measured for normalization of respiration of brain homogenates. For normalization of respirometry data, CS activity of brain homogenates was measured in respirometry samples (chamber contents) that were collected following the measurements. For assessment of mitochondrial content as independent marker, snap frozen tissue of LV, kidney, and soleus muscle was homogenized in PBS. CS activity was subsequently measured according to the manufacturer’s instructions (CS0720; Sigma-Aldrich, St. Louis, MO) and normalized per µg protein (nmol × min^−1^ × µg^−1^).

### Mitochondrial morphology

Cardiac mitochondrial number and morphology was additionally evaluated by ultrastructural analysis of the left ventricular myocardium with TEM and analyzed as previously described^108,109^. Samples were examined with a JEOL 1230 TEM (Tokyo, Japan). Myocardial mitochondria were manually traced from at least 5 calibrated images per subject at × 12,000 indirect magnification using Image J (National Institutes of Health, Bethesda, MD).

### Relative mitochondrial DNA copy number

Total genomic DNA was isolated using the Zymo Quick DNA Miniprep Plus Kit (Zymo Research Corporation, Irvine, California, USA) according to manufacturer’s instructions. Two sets of primers and probes were selected: (1) one targeting the mitochondrial encoded NADH dehydrogenase subunit 4 (MT-ND4) and (2) one targeting the nuclear encoded β-actin (ACTB), and obtained from Integrated DNA Technologies (Coralville, Iowa, USA)^110^. See Supplementary Table S3 for sequences. qPCR was performed on a ViiA 7 PCR System (Life Technologies, Carlsbad, California, USA) as described in Kilbaugh et al.^110^. Relative mitochondrial copy number was then determined by calculating the difference in cycle threshold (ΔC_t_) between mtDNA gene copy number (MT-ND4) and the internal control of nuclear DNA (nuclear encoded β-actin, ACTB). By using an internal control (the nuclear encoded β-actin), variations in sample preparation, quality, and PCR efficiency can be accounted for.

### Oxidative damage

Protein Carbonyl (PC) and 3-Nitrotyrosine (3NT) levels, biochemical markers of oxidative damage, were assessed in cortex and LV using commercially available kits (OxiSelect™ Protein Carbonyl ELISA Kit, OxiSelect™ Nitrotyrosine ELISA Kit, Cell Biolabs) according to the manufacturer´s instructions. Cortex and LV (50 mg/mL) were homogenized in PBS containing butylated hydroxytoluene (0.005%, v/v), pH 7.4. Following homogenization, the samples were centrifuged at 16,000*g* for 10 min at 4 °C and the supernatant was collected. For assessment of tissue 3NT levels, 30 µg (cortex) or 17.5 µg (LV) of protein were used in the OxiSelect™ Nitrotyrosine ELISA Kit. 3NT levels were subsequently expressed as nmol × l^−1^. Remaining undiluted supernatant was then further processed for the OxiSelect™ Protein Carbonyl ELISA Kit. It was treated with streptomycin (1%, v/v) to remove nucleic acids from the tissue homogenates, and 1 µg protein was subsequently used to measure PC levels. PC levels were expressed as nmol × mg protein^−1^.

### Statistics

Previous studies have shown that DMF increases mitochondrial respiration and content^52,55^. Therefore, the primary outcomes of this study were mitochondrial respiration and mitochondrial content of brain and heart. Based on previously published studies^52,55^ we expected a large effect size by DMF. However, given the exploratory nature of our study, the magnitude of change in mitochondrial function and content of brain and heart in response to DMF treatment was unknown before this study. Therefore, the sample size was decided based on previously published data of our laboratory demonstrating a significant decrease in cerebral mitochondrial respiration (OXPHOS_CI+II_ capacity of cortex and hippocampus) and—content (citrate synthase activity of hippocampus) following cardiac arrest with a sample size of 5 animals per group^58^. Statistical analysis was performed using GraphPad Prism (GraphPad Software, San Diego, California, USA). Normally distributed data were analyzed using ordinary one-way ANOVA (homogenous variances) or Brown–Forsythe and Welch ANOVA (non-homogenous variances). For comparisons of non-normally distributed data, Kruskal–Wallis test was applied. Tukey’s multiple comparison test with comparison of every group with each of the other groups was used to evaluate treatment with DMF. Data are presented as mean ± SD. Correlation was evaluated using Spearman’s correlation coefficient. P < 0.05 was considered to indicate statistically significant differences. Graphical illustrations were prepared using Adobe illustrator, Biorender, and the Noun project.

### Supplementary Information

Supplementary Information.

## Data Availability

The datasets used and/or analyzed during the current study are available from the corresponding author [Sarah Piel] on reasonable request.
