# Supplementary material for: Effect of dimethyl fumarate on mitochondrial metabolism in a pediatric porcine model of asphyxia-induced in-hospital cardiac arrest
Source: Sci Rep. 2024 Jun 15;14:13852. doi: 10.1038/s41598-024-64317-9 (PMC11180202; doi:10.1038/s41598-024-64317-9)
Supplement: Supplementary file 1 — Supplementary Information. [file 41598_2024_64317_MOESM1_ESM.pdf]

Effect of dimethyl fumarate on mitochondrial metabolism in a pediatric porcine model of asphyxia-induced in-hospital cardiac arrest

Sarah Piel[1,2,3,4]*, Meagan J. McManus[1,2]*, Kristina N. Heye[5], Forrest Beaulieu[6], Hossein Fazelinia[7], Joanna I. Janowska[1,2], Bryce MacTurk[1,2], Jonathan Starr[1,2], Hunter Gaudio[1,2], Nisha Patel[1,2], Marco M. Hefti[8], Martin E. Smalley[8], Jordan N. Hook[8], Neha V. Kohli[1,2], James Bruton[1,2], Thomas Hallowell[1,2], Nile Delso[1,2], Anna Roberts[1,2], Yuxi Lin [1,2], Johannes K. Ehinger[9,10,11], Michael Karlsson[12], Robert A. Berg[1,2], Ryan W. Morgan[1,2]and Todd J. Kilbaugh[1,2]

[1]Resuscitation Science Center of Emphasis, The Children's Hospital of Philadelphia, Philadelphia, USA; [2]Department of Anesthesiology and Critical Care Medicine, The Children's Hospital of Philadelphia, Philadelphia, USA; [3]Department of Cardiology, Pulmonology, and Vascular Medicine, University Hospital Düsseldorf, Medical Faculty of the Heinrich Heine University Düsseldorf, Düsseldorf, Germany; [4]CARID, Cardiovascular Research Institute Düsseldorf, Medical Faculty of the Heinrich-Heine-University Düsseldorf, Germany; [5]Division of Neurology, The Children's Hospital of Philadelphia, Philadelphia, USA; [6]Department of Pediatrics, The Children's Hospital of Philadelphia, Philadelphia, USA; [7]Proteomics Core Facility, The Children's Hospital of Philadelphia, Philadelphia, USA; [8]Department of Pathology, University of Iowa Carver College of Medicine, Iowa City, IA, USA; [9]Mitochondrial Medicine, Department of Clinical Sciences Lund, Lund University, Lund, Sweden; [10]Otorhinolaryngology, Department of Clinical Sciences Lund, Lund University, Lund, Sweden; [11]Otorhinolaryngology, Head and Neck Surgery, Skåne University Hospital, Lund, Sweden; [12]Neurosurgery, Rigshospitalet, Copenhagen, Denmark

*Contributed equally

Correspondence: Sarah Piel, PhD, Resuscitation Science Center of Emphasis, The Children's Hospital of Philadelphia, 3401 Civic Center Boulevard, Philadelphia, PA, 19104, USA. E-mail: piels@chop.edu. ORCiD: 0000-0002-1779-3532.

Table of Contents

Supplementary Table S1. Overview of molecular tissue analysis 3
Supplementary Table S2. Substrate-Uncoupler-Inhibitor Titration protocol 4
Supplementary Table S3. Specification of primer and probe nucleotide sequences 5
Supplementary Table S4. Hemodynamics and blood gas chemistry 6
Supplementary Table S5. Cardiopulmonary resuscitation characteristics 9
Supplementary Table S6. Cerebral proteomic profile 10
Supplementary Table S7. Cerebral proteomic profile - Pathway enrichment analysis 36
Supplementary Figures 54
Supplementary Figure S1 Citrate synthase activity. 54
Supplementary Figure S2 Relative mtDNA copy number. 55
Supplementary Figure S3 Mitochondrial size distribution. 56
Supplementary Figure S4 Cerebral and myocardial mitochondrial respiration. 57
Supplementary Figure S5 Mitochondrial respiration of tissue homogenates of the cortex. 58
Supplementary Figure S6 Mitochondrial respiration of tissue homogenates of the hippocampus. 59
Supplementary Figure S7 Oxidative damage. 60

Supplementary Tables

Supplementary Table S1. Overview of molecular tissue analysis

Assay/Organ	Cortex	Hippocampus	Left Ventricle	Right Ventricle	Soleus	Kidney
High-resolution respirometry	+	+	+	+	-	-
Citrate Synthase Activity	+	+	+	+	+	+
mtDNA/nDNA	+	+	+	+	+	+
EM	-	-	+	-	-	-
Proteomics	+	-	-	-	-	-

Tissues were preserved in a) isolation buffer for high-resolution respirometry of fresh tissue, b) Paraformaldehyde-Glutaraldehyde Solution (Karnovsky's Fixative) for electron microscopy (EM) and c) snap frozen on dry ice for further biomarker analysis.

Supplementary Table S2. Substrate-Uncoupler-Inhibitor Titration protocol

Agent	Sample type		
	Brain homogenate	Isolated brain mitochondria	Isolated heart mitochondria
Amplex red	10 µM	10 µM	10 µM
Horseradish peroxidase	1 U/mL	5 U/mL	5 U/mL
Superoxide dismutase	10 U/mL	50 U/mL	50 U/mL
Sample (mg per chamber)	1 mg/mL	25 µg/mL	13 µg/mL
Digitonin (mg/mL)	25 µg/mL	N/A	N/A
Malate (mM)	5 mM	5 mM	5 mM
Pyruvate (mM)	5 mM	5 mM	5 mM
ADP (mM)	1 mM	1 mM	5 mM
Glutamate (mM)	5 mM	5 mM	5 mM
Succinate (mM)	20 mM	20 mM	20 mM
Oligomycin (mg/mL)	1 µg/mL	1 µg/mL	1 µg/mL
FCCP	0.2 mM stock	0.2 mM stock	0.2 mM stock
Rotenone (mM)	2 µM	2 µM	2 µM
Antimycin (mg/mL)	1 µg/mL	1 µg/mL	1 µg/mL
Ascorbate (mM)	2 mM	2 mM	10 mM
TMPD (mM)	0.5 mM	0.5 mM	0.5 mM
Azide (mM)	40 mM	40 mM	120 mM

Mitochondrial respiration of brain homogenate and isolated brain mitochondria was evaluated using the Oxygraph-2k (Oroboros Instruments, Innsbruck, Austria) at 37°C, an active chamber volume of 2 mL, a stirrer speed of 750 RPM and measurements every 2 seconds. The SUIT protocol was performed in the below order and the final concentrations as indicated.

Supplementary Table S3. Specification of primer and probe nucleotide sequences

Target	Item Name	Sequence (5'-3')	Product
Porcine β-actin	Prime Time Primer 1	TTATGGGAGAGGTCAGGGTT	PrimeTime XL DNA Primer II
	Prime Time Primer 2	CCTCAGAAGCCAATGAGAGATG	PrimeTime XL DNA Primer II
	Prime Time Probe		PrimeTime Assay XL Probe 5' 6-FAM/ZEN/3' IBFQ
Porcine ND4	Prime Time Primer 1	ATAGCCTATCCATTCCTCATGCTTT	PrimeTime XL DNA Primer II
	Prime Time Primer 2	GTGTACTCGTTCATAGTTAGTGTTGG	PrimeTime XL DNA Primer II
	Prime Time Probe		PrimeTime Assay XL Probe 5' 6-FAM/ZEN/3' IBFQ

Supplementary Table S4. Hemodynamics and blood gas chemistry

Variable	Sham	Placebo	DMF
		Baseline Period	
Body temperature, °C	37.5 (1.3)	36.6 (0.7)	36.6 (0.6)
Heart rate, bpm	115.4 (11.3)	114.2 (11.8)	115.3 (12.2)
MAP, mmHg	58.7 (7.5)	55.5 (8.6)	57.2 (4.4)
SBP, mmHg	73.8 (14.5)	72.6 (9.9)	68.8 (7.3)
DBP, mmHg	48.1 (5.1)	44.3 (7.4)	48.3 (2.7)
CoPP, mmHg	41.4 (4.27)	35.2 (8.0)	39.3 (3.9)
PaO_2, mmHg	82.8 (10.7)	98.2 (13.6)	97.6 (11.6)
Arterial blood SO_2, %	96.6 (1.5)	97.8 (0.8)	97.8 (0.8)
$PaCO_2$, mmHg	40.8 (2.7)	41.8 (7.3)	37.2 (4.7)
$EtCO_2$, mmHg	42.8 (3.6)	44.8 (5.7)	41.5 (4.2)
		Asphyxia	
Body temperature, °C	37.6 (1.4)	36.7 (0.8)	36.5 (0.9)
Heart rate, bpm	107.8 (18.1)	101.6 (26.70)	104.8 (16.84)
MAP, mmHg	65.9 (13.4)	31.7 (10.3) [aa]	26.9 (16.3) [bb]
SBP, mmHg	82.4 (17.5)	47.5 (20.3)	48.9 (39.1)
DBP, mmHg	54.1 (12.2)	28.6 (10.1) [aa]	22.0 (10.8) [bb]
CoPP, mmHg	46.4 (11.6)	15.5 (11.2)	13.6 (18.3)
PaO_2, mmHg	88.0 (4.6)	5.8 (3.1) [aaa]	3.7 (1.5) [bbb]

Arterial blood SO_2, %	97.25 (0.50)	3.40 (2.41)	2.00 (1.00) [b]
$PaCO_2$, mmHg	35.8 (2.2)	67.8 (11.7) [aa]	78.7 (9.3) [b]
$EtCO_2$, mmHg	41.76 (3.13)	4.88 (5.98) [aaa]	8.77 (13.68)
CPR			
Body temperature, °C	37.5 (1.5)	36.5 (1.0)	36.5 (1.2)
Heart rate, bpm	107.1 (19.0)	101.2 (2.48)	100.6 (1.19)
MAP, mmHg	63.1 (16.8)	42.8 (3.3) [aa]	45.3 (3.2)
SBP, mmHg	78.9 (22.3)	84.8 (6.2)	84.9 (7.6)
DBP, mmHg	51.6 (14.8)	34.1 (3.9) [aa]	35.7 (1.9)
CoPP, mmHg	44.7 (12.6)	21.43 (2.8) [aa]	18.4 (9.6) [bb]
PaO_2, mmHg	N.S.	243.0 (112.8)	145.0 (100.2)
Arterial blood SO_2, %	N.S.	96.2 (8.5)	96.4 (3.5)
$PaCO_2$, mmHg	N.S.	41.2 (6.8)	34.4 (8.5)
$EtCO_2$, mmHg	41.41 (3.18)	33.15 (6.49) [a]	28.96 (3.62) [bb]
Post-ROSC			
Body temperature, °C	37.8 (0.6)	37.2 (1.0)	36.5 (1.3)
Heart rate, bpm	115.5 (17.6)	153.9 (22.2)	137.7 (36.1)
MAP, mmHg	57.4 (8.3)	55.3 (7.4)	65.8 (4.3)
SBP, mmHg	74.4 (11.0)	68.6 (12.5)	76.7 (5.4)
DBP, mmHg	44.7 (6.1)	47.8 (4.6)	59.4 (5.1) [bb, c]

CoPP, mmHg	37.1 (9.7)	40.1 (5.7)	46.9 (7.9)
PaO_2, mmHg	86.8 (7.0)	82.0 (8.8)	92.2 (22.6)
Arterial blood SO_2, %	97.25 (0.50)	95.25 (2.22)	95.00 (4.74)
$PaCO_2$, mmHg	39.5 (4.1)	40.5 (4.7)	41.0 (11.1)
$EtCO_2$, mmHg	43.24 (1.77)	45.69 (4.04)	47.63 (3.62)

Hemodynamics and venous blood gases were measured before randomization into the respective treatment group at baseline, during asphyxia and cardiopulmonary resuscitation (CPR) and following return of spontaneous circulation (ROSC). Three groups were statistically compared using ordinary one-way ANOVA (homogenous variances), Brown-Forsythe and Welch ANOVA (non-homogenous variances), or Kruskal Wallis test (non-parametric) whereas two group comparison was performed using student's t-test (homogenous variances), Welch's t-test (non-homogenous variances) or Mann-Whitney test (non-parametric). Corresponding letters indicating the respective group comparison: a=sham vs placebo (a=$p < 0.05$, aa=$p < 0.01$ and aaa=$p < 0.001$); b=sham vs DMF (b=$p < 0.05$, bb=$p < 0.01$ and bbb=$p < 0.001$); c=placebo vs DMF (c=$p < 0.05$, cc=$p < 0.01$ and ccc=$p < 0.001$). Data are presented as mean ± standard deviation. n=2-5. bpm = beats per minute; MAP=mean arterial pressure; SBP=systolic blood pressure; DBP=diastolic blood pressure; CoPP=coronary perfusion pressure; PaO_2=arterial oxygen pressure; SO_2=oxygen saturation; $PaCO_2$=arterial carbon diaoxide; $EtCO_2$=end-tidal carbon dioxide. N.S=not sampled.

Supplementary Table S5. Cardiopulmonary resuscitation characteristics

Variable	placebo	DMF	P value
Chest compression rate per min^{-1}	100.6(1.28)	95.65(0.21)	-
Chest compression depth, cm	33.25(5.84)	31.69(2.28	-
CPR duration, min	10(0)	11.6(2.19)	0.44
Vasopressor doses, n	4.2(1.6)	6.0(2.1)	0.37
Defibrillation attempts, n	1.0(0)	1.8(1.1)	0.44

Chest compression, depth, duration as well as required number of vasopressor doses and defibrillation attempts was measured during cardiopulmonary resuscitation (CPR). CPR characteristics were statistically compared using student's t-test (homogenous variances), Welch's t-test (non-homogenous variances) or Mann-Whitney test (non-parametric). Data are presented as mean ± standard deviation. n=2-5.

Supplementary Table S6. Cerebral proteomic profile

Group comparison	Up/Down	p-value	Log FC Difference	Genes	Protein Descriptions
Placebo vs Sham	Downregulated	0,017	-4,25	ENPP2	Ectonucleotide pyrophosphatase/phosphodiesterase 2
		0,007	-2,79	DHRS1	Dehydrogenase/reductase 1
		0,010	-1,69	SLC39A12	Solute carrier family 39 member 12
		0,023	-1,44	WIPF3	WAS/WASL interacting protein family member 3
		0,040	-1,37	AP1AR	Uncharacterized protein FAM241A
		0,024	-1,36	JCHAIN	Joining chain of multimeric IgA and IgM
		0,048	-1,29	MT2A	Metallothionein-2A
		0,007	-1,21	SDF2	Stromal cell-derived factor 2
		0,031	-1,20	C14H1orf198	Chromosome 14 C1orf198 homolog
		0,032	-1,20	KCNH3	Potassium voltage-gated channel subfamily H member 3
		0,004	-1,19	KCNT1;KCNT2;KCNT2;KCNT2;KCNT1;KCNT1;KCNT1;KCNT2;KCNT1;KCNT1;KCNT1;KCNT2	Potassium sodium-activated channel subfamily T member 1;Potassium sodium-activated channel subfamily T member 2;Potassium sodium-activated channel subfamily T member 2;Potassium sodium-activated channel subfamily T member 2;Potassium channel subfamily T member 1 isoform X5;Potassium sodium-activated channel subfamily T member 1;Potassium sodium-activated channel subfamily T member 1;Potassium sodium-activated channel subfamily T member 2;Potassium sodium-activated channel subfamily T member 1;Potassium sodium-activated channel subfamily T member 1;Potassium sodium-activated channel subfamily T member 1;Potassium sodium-activated channel subfamily T member 2
		0,040	-1,18	MTMR10	Myotubularin-related protein 10;Myotubularin related protein 10
		0,046	-1,12	OARD1	O-acyl-ADP-ribose deacylase 1;O-acetyl-ADP-ribose deacetylase 1
		0,050	-1,07	BCDIN3D	RNA methyltransferase
		0,040	-1,06	MYO16	Uncharacterized protein; Myosin motor domain-containing protein4
		0,010	-1,06	C6H19orf12	Chromosome 6 C19orf12 homolog

Supplementary Table S6. Cerebral proteomic profile

Group comparison	Up/Down	p-value	Log FC Difference	Genes	Protein Descriptions
		0,044	-1,03	MMP17	Matrix metallopeptidase 17
		0,002	-1,02	KCTD7	Potassium channel tetramerization domain containing 7
		0,033	-1,00	POMT1	Dolichyl-phosphate-mannose--protein mannosyltransferase
		0,006	-0,97	GATAD2B	GATA zinc finger domain containing 2B
		0,001	-0,96	CSDC2	Cold shock domain containing C2
		0,004	-0,95	DPP8	Dipeptidyl peptidase 8
		0,022	-0,93	SLC20A1	Phosphate transporter
		0,026	-0,87	MRPL47	Mitochondrial ribosomal protein L47
		0,011	-0,86	ATP5F1E	Uncharacterized protein
		0,040	-0,85	COX8A	Cytochrome c oxidase subunit 8A
		0,000	-0,85	MPC1	Mitochondrial pyruvate carrier
		0,000	-0,84	MICOS10	MICOS complex subunit MIC10
		0,021	-0,83	PNPLA8	Patatin like phospholipase domain containing 8;Calcium-independent phospholipase A2-gamma isoform 1
		0,046	-0,80	TUBA3E; LOC100510930;LOC100155138;LOC100155138;LOC100510930	Tubulin alpha chain
		0,037	-0,78	TBL1XR1	TBL1X receptor 1;F-box-like/WD repeat-containing protein TBL1XR1 isoform 1
		0,027	-0,77	LSM7	LSM7 homolog, U6 small nuclear RNA and mRNA degradation associated;LSM7 homolog, U6 small nuclear RNA and mRNA degradation associated;LSM7 homolog, U6 small nuclear RNA and mRNA degradation associated
		0,035	-0,76	ISCA1	Fe-S_biosyn domain-containing protein
		0,049	-0,76	TMX1	Thioredoxin-related transmembrane protein 1
		0,041	-0,75	NDUFS5	Complex I-15 kDa
		0,014	-0,72	CD5L	CD5 molecule like
		0,042	-0,71	MFSD4A	Major facilitator superfamily domain containing 4A

Supplementary Table S6. Cerebral proteomic profile

Group comparison	Up/Down	p-value	Log FC Difference	Genes	Protein Descriptions
		0,036	-0,69	TMSB4; LOC110257905;TMSB4	Thymosin beta;Thymosin beta-4
		0,039	-0,66	ATL2	Atlastin GTPase 2
		0,013	-0,66	HINT3	Histidine triad nucleotide binding protein 3
		0,002	-0,65	CD81	Tetraspanin
		0,039	-0,65	PPP2CA	Serine/threonine-protein phosphatase 2A catalytic subunit alpha isoform
		0,037	-0,64	UCK2	Uridine-cytidine kinase
		0,045	-0,64	AGPAT1	1-acyl-sn-glycerol-3-phosphate acyltransferase;1-acyl-sn-glycerol-3-phosphate acyltransferase;1-acyl-sn-glycerol-3-phosphate acyltransferase;1-acylglycerol-3-phosphate O-acyltransferase 1
		0,035	-0,64	ABHD17B	Abhydrolase domain containing 17B, depalmitoylase
		0,020	-0,63	SNRPF	Sm protein F
		0,012	-0,63	ARMC9	LisH domain-containing protein ARMC9
		0,024	-0,63	PPIA	Peptidyl-prolyl cis-trans isomerase
		0,038	-0,62	CCDC97	Coiled-coil domain containing 97
		0,006	-0,62	MPDU1	Mannose-P-dolichol utilization defect 1 protein
		0,023	-0,60	ISLR2	Immunoglobulin superfamily containing leucine rich repeat 2
		0,017	-0,60	TNFAIP8L3	TNF alpha induced protein 8 like 3
		0,034	-0,59	EBP	Emopamil binding protein (Sterol isomerase)
		0,020	-0,59	CTPS1	CTP synthase
		0,048	-0,58	GNPNAT1	Glucosamine 6-phosphate N-acetyltransferase
		0,002	-0,58	ATPAF2	ATP synthase mitochondrial F1 complex assembly factor 2
		0,016	-0,57	ARL8B	ADP ribosylation factor like GTPase 8B
		0,018	-0,57	AGPS	Alkylglycerone-phosphate synthase
		0,006	-0,56	RHOC	Ras homolog family member C
		0,049	-0,55	FDPS	Farnesyl diphosphate synthase
		0,027	-0,55	ILKAP	ILK associated serine/threonine phosphatase
		0,032	-0,54		H(+)-transporting two-sector ATPase

Supplementary Table S6. Cerebral proteomic profile

Group comparison	Up/Down	p-value	Log FC Difference	Genes	Protein Descriptions
		0,048	-0,54	G6PC3	Glucose-6-phosphatase
		0,025	-0,53	MESD	LDLR chaperone MESD
		0,045	-0,53	RIDA	2-iminobutanoate/2-iminopropanoate deaminase
		0,047	-0,53	MIF4GD	MIF4G domain containing
		0,034	-0,51	GMPPB	Mannose-1-phosphate guanyltransferase beta
		0,016	-0,51	EIF2A	Eukaryotic translation initiation factor 2A
		0,007	-0,50	HDHD5	Haloacid dehalogenase like hydrolase domain containing 5
		0,036	-0,49	RPL15	Ribosomal protein L15
		0,031	-0,48	CST3	Cystatin C
		0,029	-0,48	RPL11	60S ribosomal protein L11
		0,030	-0,47	USP5	Ubiquitin carboxyl-terminal hydrolase
		0,021	-0,47	VPS41	Vacuolar protein sorting-associated protein 41 homolog
		0,038	-0,46	NIPSNAP1	NIPSNAP domain-containing protein
		0,048	-0,46	DYNC1I2	Dynein cytoplasmic 1 intermediate chain 2
		0,018	-0,45	SUMO2	Small ubiquitin-related modifier 2;Small ubiquitin-related modifier;Small ubiquitin-related modifier 2
		0,012	-0,45	RPL7L1	60S ribosomal protein L7-like 1
		0,014	-0,44	RBBP9	RB binding protein 9, serine hydrolase;Putative hydrolase RBBP9
		0,036	-0,44	HEXA	Beta-hexosaminidase
		0,002	-0,44	RACK1	Receptor of activated protein C kinase 1
		0,013	-0,43	STX6	Syntaxin 6
		0,004	-0,43	DNAJC19	DnaJ heat shock protein family (Hsp40) member C19
		0,034	-0,42	LYPLA1	Lysophospholipase 1;Acyl-protein thioesterase 1 isoform 1
		0,047	-0,41		HMA domain-containing protein
		0,016	-0,41	NDUFB9	Complex I-B22
		0,043	-0,41	TNR	Tenascin R

Supplementary Table S6. Cerebral proteomic profile

Group comparison	Up/Down	p-value	Log FC Difference	Genes	Protein Descriptions
		0,004	-0,41	LRCH2	Leucine rich repeats and calponin homology domain containing 2
		0,024	-0,40	NDUFA11	Complex I-B14.7
		0,020	-0,39	GOT2	Aspartate aminotransferase;Aspartate aminotransferase, mitochondrial
		0,007	-0,39	AGPAT5	1-acylglycerol-3-phosphate O-acyltransferase 5
		0,027	-0,39	SLC25A3	Phosphate carrier protein, mitochondrial;Phosphate carrier protein, mitochondrial;Phosphate carrier protein, mitochondrial
		0,039	-0,38	RRP12	Ribosomal RNA processing 12 homolog
		0,032	-0,37	GMPPA	Mannose-1-phosphate guanyltransferase alpha
		0,027	-0,37	EGLN1	Egl-9 family hypoxia inducible factor 1
		0,016	-0,36	SSR1	Signal sequence receptor subunit alpha
		0,019	-0,35	GFUS	GDP-4-keto-6-deoxy-D-mannose-3,5-epimerase-4-reductase;GDP-4-keto-6-deoxy-D-mannose-3,5-epimerase-4-reductase
		0,021	-0,35	FAM120B	Constitutive coactivator of peroxisome proliferator-activated receptor gamma isoform a
		0,021	-0,35	RPL18	60S ribosomal protein L18
		0,025	-0,34	UBE2I	UBC core domain-containing protein
		0,045	-0,34	MMUT	Methylmalonyl-CoA isomerase;Methylmalonyl-CoA isomerase;Methylmalonyl-CoA mutase, mitochondrial
		0,020	-0,33	RPL27A	60S ribosomal protein L27a
		0,025	-0,32	COX5A	Cytochrome c oxidase polypeptide Va
		0,020	-0,32	DPP3	Dipeptidyl peptidase 3
		0,019	-0,32	SCCPDH	Sacchrp_dh_NADP domain-containing protein
		0,047	-0,31	HNRNPL	Uncharacterized protein
		0,027	-0,31	RPS23	40S ribosomal protein S23
		0,012	-0,31	GNPDA1	Glucosamine-6-phosphate isomerase
		0,042	-0,30	NIPSNAP3A; LOC100739365	Protein NipSnap homolog 3A isoform 1
		0,004	-0,30	KLHL26	Kelch like family member 26

Supplementary Table S6. Cerebral proteomic profile

Group comparison	Up/Down	p-value	Log FC Difference	Genes	Protein Descriptions
		0,006	-0,29	PCYOX1L	Prenylcysteine oxidase 1 like
		0,007	-0,29	LOC100302368;	Trypsinogen isoform X1;Trypsin
		0,038	-0,28	MIGA1	Mitoguardin 1
		0,021	-0,28	AKT3	Non-specific serine/threonine protein kinase
		0,030	-0,27	NDUFV1	NADH dehydrogenase [ubiquinone] flavoprotein 1, mitochondrial
		0,030	-0,27	ATXN10	Ataxin 10
		0,020	-0,27	ALDH1L2	Formyltetrahydrofolate dehydrogenase
		0,032	-0,26	SFXN4	Sideroflexin 4
		0,029	-0,26	RPL7A	60S ribosomal protein L7a
		0,043	-0,26	EFL1	Elongation factor like GTPase 1
		0,025	-0,26	DLAT	Acetyltransferase component of pyruvate dehydrogenase complex
		0,042	-0,25	RPL24	TRASH domain-containing protein
		0,049	-0,25	ACTR1B	Actin related protein 1B
		0,044	-0,24	OLA1	Obg-like ATPase 1
		0,012	-0,24	CRAT	Carnitine O-acetyltransferase
		0,042	-0,24	SAR1A	GTP-binding protein SAR1a
		0,039	-0,24	RPS2	40S ribosomal protein S2
		0,023	-0,23	TBRG4	FAST kinase domain-containing protein 4
		0,049	-0,22	SSRP1	FACT complex subunit SSRP1
		0,027	-0,22	LONP2	Lon protease homolog 2, peroxisomal;Lon protease homolog 2, peroxisomal
		0,017	-0,21	U2AF2	U2 snRNP auxiliary factor large subunit
		0,037	-0,20	HSD17B10	Hydroxysteroid 17-beta dehydrogenase 10
		0,016	-0,17	STK38L	Serine/threonine kinase 38 like
		0,045	-0,17	RPS12	40S ribosomal protein S12
		0,047	-0,17	SLC25A11	Mitochondrial 2-oxoglutarate/malate carrier protein isoform 1
		0,039	-0,17	LANCL1	LanC like 1
		0,044	-0,17	PTGES3	Prostaglandin E synthase 3

Supplementary Table S6. Cerebral proteomic profile

Group comparison	Up/Down	p-value	Log FC Difference	Genes	Protein Descriptions
		0,047	-0,16	PCBP2	Uncharacterized protein
		0,039	-0,09	HSPA8	Uncharacterized protein
	Upregulated	0,041	0,11	PSMD1	26S proteasome non-ATPase regulatory subunit 1
		0,005	0,12	BCS1L	BCS1-like protein
		0,023	0,12	ADD3	Adducin 3
		0,045	0,13	USP7	Ubiquitin carboxyl-terminal hydrolase 7
		0,024	0,15	SPTBN4	Spectrin beta, non-erythrocytic 4
		0,011	0,15	FARSB	Phenylalanyl-tRNA synthetase beta subunit
		0,014	0,16	DNAJA3	DnaJ homolog subfamily A member 3, mitochondrial isoform 1;Uncharacterized protein;Uncharacterized protein
		0,004	0,18	DNAJC11	DnaJ heat shock protein family (Hsp40) member C11
		0,028	0,18	ARHGAP5	Rho GTPase activating protein 5
		0,046	0,19	RAB14	Ras-related protein Rab-14
		0,031	0,19	USP10	Ubiquitin carboxyl-terminal hydrolase
		0,050	0,21	PIK3R1	Phosphatidylinositol 3-kinase 85 kDa regulatory subunit alpha
		0,037	0,22	EDC4	Enhancer of mRNA-decapping protein 4
		0,038	0,23	NSFL1C	NSFL1 cofactor p47
		0,019	0,23	DOCK9	Dedicator of cytokinesis 9
		0,025	0,24	TRIP12	E3 ubiquitin-protein ligase TRIP12
		0,039	0,26	COMMD2	COMM domain containing 2
		0,045	0,27	FHIP2A	Family with sequence similarity 160 member B1
		0,033	0,28	RMDN3	Regulator of microtubule dynamics 3
		0,033	0,28	AMER2	APC membrane recruitment protein 2
		0,044	0,28	FMR1	Synaptic functional regulator FMR1
		0,011	0,28	SNRNP70	U1 small nuclear ribonucleoprotein 70 kDa
		0,001	0,29	PDK3	Protein-serine/threonine kinase
		0,046	0,31	UBXN6	UBX domain protein 6

Supplementary Table S6. Cerebral proteomic profile

Group comparison	Up/Down	p-value	Log FC Difference	Genes	Protein Descriptions
		0,016	0,31	HERC1	HECT and RLD domain containing E3 ubiquitin protein ligase family member 1
		0,008	0,31	PDP1	Pyruvate dehyrogenase phosphatase catalytic subunit 1
		0,021	0,31	TUBB4B	Tubulin beta chain
		0,049	0,31	CCDC93	Coiled-coil domain-containing protein 93
		0,025	0,32	LRRFIP2	LRR binding FLII interacting protein 2
		0,032	0,32	HDAC6	Histone deacetylase 6
		0,036	0,32	CLIP1	CAP-Gly domain containing linker protein 1
		0,045	0,32	CEP170	Centrosomal protein 170
		0,005	0,34	DNAJB4	DnaJ heat shock protein family (Hsp40) member B4
		0,041	0,34	GUCY1B1	Guanylate cyclase
		0,019	0,35	NLN	Neurolysin, mitochondrial;Neurolysin, mitochondrial;Neurolysin, mitochondrial;Neurolysin, mitochondrial;Neurolysin, mitochondrial;Neurolysin, mitochondrial;Neurolysin, mitochondrial;Isoform 2 of Neurolysin, mitochondrial;Isoform 3 of Neurolysin, mitochondrial
		0,035	0,35	SNX30	Sorting nexin-30
		0,008	0,36	BORCS5	BLOC-1-related complex subunit 5
		0,020	0,36	FADD	FAS-associated death domain protein
		0,015	0,37	CAVIN1	Caveolae associated protein 1
		0,034	0,38	MCRIP1	MAPK regulated corepressor interacting protein 1
		0,014	0,38	RABEP1	Rabaptin, RAB GTPase binding effector protein 1;Rabaptin, RAB GTPase binding effector protein 1
		0,050	0,39	KIFAP3	Kinesin associated protein 3
		0,014	0,39	VPS13D	Vacuolar protein sorting 13 homolog D
		0,019	0,39	SRSF2	Serine/arginine-rich splicing factor 2

Supplementary Table S6. Cerebral proteomic profile

Group comparison	Up/Down	p-value	Log FC Difference	Genes	Protein Descriptions
		0,039	0,42	TAP1	Transporter 1, ATP binding cassette subfamily B member;Transporter 1, ATP binding cassette subfamily B member
		0,019	0,43	PTBP3	Polypyrimidine tract binding protein 3
		0,026	0,43	PTP4A1	Protein tyrosine phosphatase type IVA 1
		0,003	0,44	THBD	Thrombomodulin
		0,045	0,46	CLPP	ATP-dependent Clp protease proteolytic subunit
		0,009	0,48	MRPL48	39S ribosomal protein L48, mitochondrial;Mitochondrial ribosomal protein L48
		0,040	0,49	NSMAF	Neutral sphingomyelinase activation associated factor
		0,012	0,50	GUCY1A1	Guanylate cyclase
		0,018	0,51	LAMTOR5	Ragulator complex protein LAMTOR5
		0,012	0,51	CCDC177	Coiled-coil domain containing 177
		0,029	0,53	LSM4	U6 snRNA-associated Sm-like protein LSm4
		0,014	0,54	DPYD	Dihydropyrimidine dehydrogenase [NADP(+)]
		0,036	0,55	SMARCE1	SWI/SNF related, matrix associated, actin dependent regulator of chromatin, subfamily e, member 1;SWI/SNF related, matrix associated, actin dependent regulator of chromatin, subfamily e, member 1;SWI/SNF related, matrix associated, actin dependent regulator of chromatin, subfamily e, member 1;SWI/SNF related, matrix associated, actin dependent regulator of chromatin, subfamily e, member 1;SWI/SNF related, matrix associated, actin dependent regulator of chromatin, subfamily e, member 1
		0,049	0,55	CAMK1D	Calcium/calmodulin dependent protein kinase ID
		0,029	0,56	F8A1	Factor VIII intron 22 protein
		0,047	0,56	SLMAP	Sarcolemma associated protein
		0,014	0,58	RBP1	FABP domain-containing protein
		0,049	0,60	AKAP9	A-kinase anchoring protein 9

Supplementary Table S6. Cerebral proteomic profile

Group comparison	Up/Down	p-value	Log FC Difference	Genes	Protein Descriptions
		0,041	0,62	MRPL22	39S ribosomal protein L22, mitochondrial;39S ribosomal protein L22, mitochondrial;39S ribosomal protein L22, mitochondrial;39S ribosomal protein L22, mitochondrial;39S ribosomal protein L22, mitochondrial
		0,033	0,62	PSMD9	26S proteasome non-ATPase regulatory subunit 9
		0,022	0,63	CCDC124	Coiled-coil domain containing 124
		0,011	0,63	MINDY2	Ubiquitin carboxyl-terminal hydrolase
		0,034	0,65	SMPD4	Sphingomyelin phosphodiesterase 4
		0,015	0,65	SLC39A6	Solute carrier family 39 member 6;Solute carrier family 39 (Zinc transporter), member 6 tv1
		0,023	0,66	CREB1	Cyclic AMP-responsive element-binding protein 1 isoform B;cAMP responsive element binding protein 1;cAMP responsive element binding protein 1;cAMP responsive element binding protein 1;cAMP responsive element binding protein 1
		0,042	0,71	BCL11A	BAF chromatin remodeling complex subunit BCL11A;BAF chromatin remodeling complex subunit BCL11A;BAF chromatin remodeling complex subunit BCL11A;BAF chromatin remodeling complex subunit BCL11A;B-cell CLL/lymphoma 11A
		0,007	0,71	KBTBD6	Kelch repeat and BTB domain-containing protein 6
		0,019	0,74	PRELP	Prolargin
		0,006	0,76	CCDC149	Coiled-coil domain containing 149
		0,040	0,77	FAM171A2	Family with sequence similarity 171 member A2
		0,010	0,78	SPRED1	Sprouty related EVH1 domain containing 1
		0,028	0,83	ETNK1	Ethanolamine kinase 1
		0,017	0,85	STN1	CST complex subunit STN1
		0,021	0,87	AZI2	5-azacytidine induced 2
		0,011	0,89	PACSIN3	Protein kinase C and casein kinase substrate in neurons 3
		0,038	0,93	CELSR2	Cadherin EGF LAG seven-pass G-type receptor 2

Supplementary Table S6. Cerebral proteomic profile

Group comparison	Up/Down	p-value	Log FC Difference	Genes	Protein Descriptions
		0,018	0,95	LMBRD2	LMBR1 domain containing 2
		0,017	0,97	SPATS2L	Spermatogenesis associated serine rich 2 like
		0,026	0,98	TSC22D1	TSC22 domain family member 1
		0,005	1,02	HTR1F	5-hydroxytryptamine receptor 1F
		0,026	1,04	C6H1orf50	Chromosome 6 C1orf50 homolog
		0,000	1,11	POLR2G	RNA polymerase II subunit G;DNA-directed RNA polymerase II subunit RPB7;DNA-directed RNA polymerase II subunit RPB7
		0,017	1,13	RPIA	Ribose-5-phosphate isomerase
		0,034	1,16	ALDH1A1	Aldehyde dehydrogenase 1 family member A1
		0,044	1,37	IGHMBP2	Immunoglobulin mu DNA binding protein 2
		0,033	1,44	COLEC12	Collectin-12
		0,004	1,82	SDCBP	Syndecan binding protein
		0,008	2,29	LOC102162880	Uncharacterized protein
DMF vs Placebo	Upregulated	0,050	0,32	RPS18	40S ribosomal protein S18
		0,049	0,16	PDHA1	Pyruvate dehydrogenase E1 component subunit alpha, somatic form, mitochondrial (Fragment)
		0,049	0,37	GLRX3	Glutaredoxin 3
		0,049	0,57	SNRPF	Sm protein F
		0,049	0,33	SSR1	Signal sequence receptor subunit alpha
		0,048	0,51	SRRM1	Serine and arginine repetitive matrix 1
		0,047	0,23	TCF25	Transcription factor 25
		0,047	0,30	LZTFL1	Leucine zipper transcription factor-like protein 1
		0,046	0,25	RALB	Small monomeric GTPase
		0,045	0,88	SLC20A1	Phosphate transporter
		0,045	0,28	GNB4	G protein subunit beta 4
		0,045	0,27	TBC1D10B	TBC1 domain family member 10B
		0,045	0,39	FNTB	Protein farnesyltransferase subunit beta
		0,044	0,20	CALU	Calumenin
		0,044	3,81	TSPAN13	Tetraspanin-13

Supplementary Table S6. Cerebral proteomic profile

Group comparison	Up/Down	p-value	Log FC Difference	Genes	Protein Descriptions
		0,044	0,64	SRI	Sorcin
		0,044	0,07	USP9X	Ubiquitinyl hydrolase 1
		0,043	0,71	CTSF	Cathepsin F
		0,042	0,14	GMFB	Glia maturation factor
		0,042	1,28	IGSF11	Immunoglobulin superfamily member 11
		0,042	0,38	ORMDL1	ORM1-like protein
		0,042	0,35	TWF2	Twinfilin actin binding protein 2
		0,042	0,79	LY6E	LY6E;UPAR/Ly6 domain-containing protein;UPAR/Ly6 domain-containing protein
		0,041	0,83	CNOT4	CCR4-NOT transcription complex subunit 4
		0,041	0,41	VPS28	Vacuolar protein sorting-associated protein 28 homolog
		0,041	0,14	NIT2	Omega-amidase NIT2
		0,040	0,33	KIAA1841	KIAA1841
		0,040	0,16	SYT12	Synaptotagmin 12
		0,040	0,66	CD9	Tetraspanin
		0,039	0,25	PAK2	Non-specific serine/threonine protein kinase
		0,039	1,61	FCHO2	F-BAR domain only protein 2;FCH and mu domain containing endocytic adaptor 2;F-BAR domain only protein 2;F-BAR domain only protein 2;F-BAR domain only protein 2;F-BAR domain only protein 2
		0,039	0,16	PREP	Prolyl endopeptidase
		0,039	0,10	RBBP4	Histone-binding protein RBBP4
		0,039	0,10	CNTN1	Contactin 1
		0,039	0,52	KBTBD3	Kelch repeat and BTB domain containing 3
		0,038	0,23	BZW1	Basic leucine zipper and W2 domains 1;Basic leucine zipper and W2 domains 1;Basic leucine zipper and W2 domain-containing protein 1
		0,038	0,13	ADH5	S-(hydroxymethyl)glutathione dehydrogenase
		0,038	0,34	GRIK2	Glutamate receptor
		0,038	0,22	NAMPT	Nicotinamide phosphoribosyltransferase

Supplementary Table S6. Cerebral proteomic profile

Group comparison	Up/Down	p-value	Log FC Difference	Genes	Protein Descriptions
		0,038	0,18	COPG1	Coatomer subunit gamma
		0,037	0,99	UBE2N	UBC core domain-containing protein
		0,037	0,21	ARPC5	Actin-related protein 2/3 complex subunit 5
		0,037	0,43	COX7C	Cytochrome c oxidase subunit 7C, mitochondrial
		0,037	0,36	SLC6A9	Transporter
		0,037	1,58	BCDIN3D	RNA methyltransferase
		0,037	0,54	MSH6	DNA mismatch repair protein
		0,037	0,23	TM9SF3	Transmembrane 9 superfamily member
		0,036	0,25	AKT3	Non-specific serine/threonine protein kinase
		0,036	0,19	STIP1	Stress induced phosphoprotein 1
		0,035	0,30	RPL34	60S ribosomal protein L34
		0,035	0,51	OXR1	Oxidation resistance 1
		0,035	0,38	RPS11	40S ribosomal protein S11
		0,034	0,37	MAPRE3	Microtubule associated protein RP/EB family member 3
		0,034	0,61	ATP5F1E	Uncharacterized protein
		0,034	0,26	SCCPDH	Sacchrp_dh_NADP domain-containing protein
		0,033	0,74	FKBP5	Peptidylprolyl isomerase
		0,033	0,28	ALDH2	Aldehyde dehydrogenase, mitochondrial;Aldehyde dehydrogenase, mitochondrial
		0,033	0,63	PPAT	Amidophosphoribosyltransferase
		0,032	0,40	MOB2	MOB kinase activator 2
		0,032	0,15	DYNC1LI1	Dynein light intermediate chain
		0,032	0,43	MAP1LC3B	Uncharacterized protein;Uncharacterized protein;Microtubule-associated protein 1 light chain 3 beta
		0,031	0,33	NDUFB9	Complex I-B22
		0,031	0,24	DBNL	Drebrin like
		0,031	0,28	BCAT1	Branched-chain-amino-acid aminotransferase

Supplementary Table S6. Cerebral proteomic profile

Group comparison	Up/Down	p-value	Log FC Difference	Genes	Protein Descriptions
		0,030	0,93	PNPLA8	Patatin like phospholipase domain containing 8;Calcium-independent phospholipase A2-gamma isoform 1
		0,030	0,52	RPS19	40S ribosomal protein S19;40S ribosomal protein S19;40S ribosomal protein S19 (Fragment)
		0,030	0,53	ILKAP	ILK associated serine/threonine phosphatase
		0,030	0,67	MICOS10	MICOS complex subunit MIC10
		0,030	0,37	TIAL1	Uncharacterized protein
		0,029	0,27	SLC2A3	Solute carrier family 2, facilitated glucose transporter member 3;Solute carrier family 2, facilitated glucose transporter member 3
		0,029	0,78	ISCA1	Fe-S_biosyn domain-containing protein
		0,029	0,48	BOLA1	BolA family member 1
		0,028	0,21	SNX3	Sorting nexin-3
		0,027	0,88	CMC1	COX assembly mitochondrial protein
		0,027	0,35	FTO	Alpha-ketoglutarate-dependent dioxygenase FTO
		0,027	1,21	CHMP2A	Charged multivesicular body protein 2A
		0,027	0,23	CADM2	Cell adhesion molecule 2
		0,027	1,10		Ig-like domain-containing protein
		0,026	0,63	CLTB	Clathrin light chain
		0,026	0,46	COX6C	Cytochrome c oxidase subunit 6C
		0,026	0,92	RNF126	RING-type E3 ubiquitin transferase
		0,026	0,43	RPL13A	60S ribosomal protein L13a
		0,025	0,35	DYNC1I2	Dynein cytoplasmic 1 intermediate chain 2
		0,025	0,37	PSMD14	26S proteasome regulatory subunit RPN11
		0,025	0,37	PALM2AKAP2	AKAP2_C domain-containing protein
		0,024	2,42	PEAK1	Pseudopodium enriched atypical kinase 1
		0,024	0,61	RBBP9	RB binding protein 9, serine hydrolase;Putative hydrolase RBBP9
		0,023	0,33	LSS	Terpene cyclase/mutase family member
		0,022	0,24	SGTA	Small glutamine rich tetratricopeptide repeat containing alpha

Supplementary Table S6. Cerebral proteomic profile

Group comparison	Up/Down	p-value	Log FC Difference	Genes	Protein Descriptions
		0,022	1,21	ARGLU1	Arginine and glutamate rich 1
		0,022	0,72	CRHBP	Corticotropin-releasing factor-binding protein
		0,022	0,45	NDUFA11	NADH dehydrogenase [ubiquinone] 1 alpha subcomplex subunit 11; Complex I-B14.7
		0,022	0,98	JAM3	Junctional adhesion molecule 3
		0,020	0,36		HMA domain-containing protein
		0,019	0,19	CRAT	Carnitine O-acetyltransferase
		0,019	0,35	NDUFC2	NADH dehydrogenase [ubiquinone] 1 subunit C2
		0,019	0,28	ARF4	ADP ribosylation factor 4
		0,018	0,73	ATL2	Atlastin GTPase 2
		0,018	0,26	HSD17B10	Hydroxysteroid 17-beta dehydrogenase 10
		0,018	0,41	VTA1	Vesicle trafficking 1
		0,017	1,08	SENP8	SUMO peptidase family member, NEDD8 specific
		0,017	1,31	CSMD2	CUB and Sushi multiple domains 2
		0,017	0,43	EMC2	ER membrane protein complex subunit 2
		0,016	0,26	RPLP0	60S acidic ribosomal protein P0
		0,016	0,40	PHKG2	Phosphorylase kinase
		0,016	0,21	HADHA	Trifunctional enzyme subunit alpha, mitochondrial
		0,015	0,28	NEFM	160 kDa neurofilament protein
		0,015	0,25	SHROOM2	Uncharacterized protein
		0,015	0,70	UBE2V2	Ubiquitin conjugating enzyme E2 V2
		0,014	0,58	HK2	Hexokinase;Hexokinase;Hexokinase-2
		0,014	0,19	CPT1A	Carnitine O-palmitoyltransferase
		0,014	0,38	ACSL4	Acyl-CoA synthetase long chain family member 4
		0,014	0,20	RPS2	40S ribosomal protein S2
		0,014	1,05	DHCR7	7-dehydrocholesterol reductase
		0,014	1,09	ATP4A	Potassium-transporting ATPase alpha chain 1
		0,013	0,75	TSPAN7	Tetraspanin
		0,013	0,34	RACK1	Receptor of activated protein C kinase 1

Supplementary Table S6. Cerebral proteomic profile

Group comparison	Up/Down	p-value	Log FC Difference	Genes	Protein Descriptions
		0,013	0,17	UMPS	OMPdecase
		0,012	0,27	SMPD3	Sphingomyelin phosphodiesterase
		0,012	0,21	CTSD	Cathepsin D
		0,012	0,18	SEPTIN9	Septin 9
		0,012	0,74	DESI1	Desumoylating isopeptidase 1
		0,011	0,34	SLC4A7	Anion exchange protein
		0,010	0,40	ALDH1B1	Aldehyde dehydrogenase 1 family member B1
		0,010	0,86	SEPTIN8	Septin
		0,010	0,23	NAA15	N-alpha-acetyltransferase 15, NatA auxiliary subunit
		0,010	1,67	UNC119	Protein unc-119 homolog A
		0,010	0,34	RPL35A;RPL35A;LOC100519675	60S ribosomal protein L35a
		0,010	0,34	ZC3H7B	Zinc finger CCCH domain-containing protein 7B
		0,009	0,59	SOD1	Superoxide dismutase [Cu-Zn]
		0,009	0,34	HSD17B12	Hydroxysteroid 17-beta dehydrogenase 12
		0,009	0,99	SMAP1	Small ArfGAP 1
		0,008	0,69	RPL11	60S ribosomal protein L11
		0,008	0,29	GNPDA1	Glucosamine-6-phosphate isomerase
		0,008	1,26	C14H1orf198	Chromosome 14 C1orf198 homolog
		0,008	0,48	VPS41	Vacuolar protein sorting-associated protein 41 homolog
		0,007	0,37	COX5A	Cytochrome c oxidase polypeptide Va
		0,007	0,12	EIF4G3	Eukaryotic translation initiation factor 4 gamma 3
		0,006	0,21	PGK1	Phosphoglycerate kinase;Phosphoglycerate kinase 1
		0,005	0,53	RIDA	2-iminobutanoate/2-iminopropanoate deaminase
		0,005	0,59	RPL7L1	60S ribosomal protein L7-like 1
		0,005	0,10	PGM1	Phosphoglucomutase 1
		0,005	0,72	CRYL1	Lambda-crystallin homolog
		0,004	0,97	HM13	Histocompatibility minor 13

Supplementary Table S6. Cerebral proteomic profile

Group comparison	Up/Down	p-value	Log FC Difference	Genes	Protein Descriptions
		0,004	0,30	RPL27A	60S ribosomal protein L27a
		0,004	0,47	PRDX1	Peroxiredoxin-1
		0,003	0,64	TMX1	Thioredoxin-related transmembrane protein 1
		0,003	0,59	FUT9	Fucosyltransferase 9
		0,003	1,60	APAF1	Apoptotic peptidase activating factor 1;Apoptotic protease-activating factor 1;Apoptotic protease-activating factor 1;Apoptotic protease-activating factor 1
		0,002	1,42	SLC39A12	Solute carrier family 39 member 12
		0,001	0,24	OLA1	Obg-like ATPase 1
		0,001	0,23	GLO1	Lactoylglutathione lyase
	Downregulated	0,038	-1,12	CWC22	CWC22 spliceosome associated protein homolog
		0,009	-0,53	SLC39A6	Solute carrier family 39 member 6;Solute carrier family 39 (Zinc transporter), member 6 tv1
		0,031	-0,16	VPS36	Vacuolar protein-sorting-associated protein 36
		0,011	-0,71	TSC22D1	TSC22 domain family member 1
		0,025	-0,48	THBD	Thrombomodulin
		0,007	-0,31	HDAC2	Histone deacetylase 2
		0,050	-0,29	TRAPPC12	Trafficking protein particle complex 12
		0,008	-0,34	ANXA11	Annexin
		0,036	-0,23	ABCF1	ATP-binding cassette sub-family F member 1
		0,019	-0,27	KCTD12	Potassium channel tetramerization domain containing 12
		0,013	-0,53	SYN3	Uncharacterized protein
		0,004	-1,27	MAP2	Microtubule-associated protein
		0,021	-1,00	GALNT16	Polypeptide N-acetylgalactosaminyltransferase
		0,000	-0,64	ELFN1	Extracellular leucine rich repeat and fibronectin type III domain containing 1
		0,003	-0,43	NCKAP1L	NCK associated protein 1 like
		0,003	-0,99	POLR2G	RNA polymerase II subunit G;DNA-directed RNA polymerase II subunit RPB7;DNA-directed RNA polymerase II subunit RPB7

Supplementary Table S6. Cerebral proteomic profile

Group comparison	Up/Down	p-value	Log FC Difference	Genes	Protein Descriptions
		0,044	-1,46	FHL2	Four and a half LIM domains 2
		0,008	-1,43	GTF2H1	General transcription factor IIH subunit 1
		0,003	-1,27	HTR1F	5-hydroxytryptamine receptor 1F
		0,006	-0,38	BIRC6	Baculoviral IAP repeat containing 6
		0,003	-0,95	AZI2	5-azacytidine induced 2
		0,001	-0,40	SNX30	Sorting nexin-30
		0,046	-1,56	PODN	Acetyl-CoA C-myristoyltransferase
		0,048	-0,57	TCOF1	Treacle ribosome biogenesis factor 1
		0,006	-1,54	GOSR1	Golgi SNAP receptor complex member 1;28 kDa Golgi SNARE protein;Golgi SNAP receptor complex member 1;28 kDa Golgi SNARE protein;Golgi SNAP receptor complex member 1
		0,048	-0,25	F8A1	Factor VIII intron 22 protein
		0,040	-0,40	GRID1	Glutamate receptor
		0,029	-0,35	GUCY1A1	Guanylate cyclase
		0,020	-0,25	TRAPPC2	Trafficking protein particle complex subunit 2
		0,032	-0,32	SYNPR	Synaptoporin
		0,029	-0,35	SLC6A7	Transporter
		0,024	-0,28	CDIPT	CDP-diacylglycerol--inositol 3-phosphatidyltransferase
		0,002	-1,08	NFKB1	Nuclear factor NF-kappa-B p105 subunit
		0,044	-0,38	PLXNB1	Plexin B1
		0,005	-1,46	CSTF1	Cleavage stimulation factor 50 kDa subunit
		0,039	-0,41	HNRNPLL	Heterogeneous nuclear ribonucleoprotein L like
		0,006	-0,52	ELMOD1	ELMO domain containing 1
		0,049	-0,23	ABHD11	Abhydrolase domain containing 11
		0,027	-0,30	SPG7	SPG7 matrix AAA peptidase subunit, paraplegin;Paraplegin
		0,016	-0,32	WDR54	WD repeat domain 54
		0,019	-0,13	FARSB	Phenylalanyl-tRNA synthetase beta subunit

Supplementary Table S6. Cerebral proteomic profile

Group comparison	Up/Down	p-value	Log FC Difference	Genes	Protein Descriptions
		0,044	-0,30	GPSM1	G protein signaling modulator 1;G-protein-signaling modulator 1;G protein signaling modulator 1
		0,003	-0,57	BEGAIN	Brain enriched guanylate kinase associated
		0,023	-0,86	PATL1	PAT1 homolog 1, processing body mRNA decay factor;PAT1 homolog 1, processing body mRNA decay factor;PAT1 homolog 1, processing body mRNA decay factor
		0,036	-2,04	TTC5	Tetratricopeptide repeat domain 5
		0,037	-0,91	MSRB2	L-methionine (R)-S-oxide reductase;L-methionine (R)-S-oxide reductase;Peptide-methionine (R)-S-oxide reductase
		0,012	-0,40	SRP72	Signal recognition particle subunit SRP72
		0,047	-0,22	AAMDC	Uncharacterized protein
		0,049	-0,46	TOMM40L	Translocase of outer mitochondrial membrane 40 like
		0,046	-0,79	SEL1L	SEL1L adaptor subunit of ERAD E3 ubiquitin ligase
		0,015	-1,11	JAKMIP3	Janus kinase and microtubule interacting protein 3
		0,025	-0,27	AHCY	Adenosylhomocysteinase
		0,037	-0,98	WNK3	Non-specific serine/threonine protein kinase
		0,049	-0,33	VPS13D	Vacuolar protein sorting 13 homolog D
		0,033	-0,30	FMR1	Synaptic functional regulator FMR1
		0,031	-0,74	ISCU	Iron-sulfur cluster assembly enzyme
		0,049	-0,25	MYH10	Uncharacterized protein
		0,046	-0,61	SCO1	Synthesis of cytochrome C oxidase 1
		0,022	-0,14	ADGRL1	Adhesion G protein-coupled receptor L1
		0,029	-0,33	STAT3	Signal transducer and activator of transcription 3
		0,038	-0,35	PPM1A	Protein-serine/threonine phosphatase
		0,045	-0,31	GEMIN5	Gem nuclear organelle associated protein 5
		0,011	-0,55	LRRC73	Leucine rich repeat containing 73
		0,030	-0,65	TBPL1	TATA box-binding protein-like 1
		0,030	-0,24	PLEKHA5	Pleckstrin homology domain containing A5

Supplementary Table S6. Cerebral proteomic profile

Group comparison	Up/Down	p-value	Log FC Difference	Genes	Protein Descriptions
		0,029	-0,68	PTPRD	Protein-tyrosine-phosphatase
		0,026	-0,81	ETNK1	Ethanolamine kinase 1
		0,046	-0,82	LMNA	Prelamin-A/C
		0,049	-0,16	DAB2IP	DAB2 interacting protein
		0,038	-0,42	PCBD1	4a-hydroxytetrahydrobiopterin dehydratase
		0,018	-0,36	TTC9	Tetratricopeptide repeat domain 9
		0,004	-1,05	SDC4	Syndecan;Syndecan;Syndecan-4
		0,044	-2,47	CD300A	CMRF35-like molecule 8 isoform 1
		0,037	-0,84	RING1	E3 ubiquitin-protein ligase RING1
		0,034	-0,23	POR	NADPH--cytochrome P450 reductase
		0,021	-0,18	PITHD1	PITH domain containing 1
		0,034	-0,40	SPATA5	Vesicle-fusing ATPase
		0,032	-0,35	SNRPD1	Small nuclear ribonucleoprotein Sm D1
		0,031	-0,30	STXBP5L	Syntaxin binding protein 5 like
		0,046	-0,27	RABEP1	Rabaptin, RAB GTPase binding effector protein 1;Rabaptin, RAB GTPase binding effector protein 1
		0,012	-0,37	HMGN1	Uncharacterized protein
		0,004	-0,35	PNPO	Pyridoxal 5'-phosphate synthase
		0,012	-0,63	KLHL25	Ectoderm-neural cortex protein 2
		0,048	-0,20	PIK3R1	Phosphatidylinositol 3-kinase 85 kDa regulatory subunit alpha
		0,035	-0,23	EFR3B	EFR3 homolog B
		0,036	-0,38	DGKH	Diacylglycerol kinase
		0,007	-0,40	GABRB3	Gamma-aminobutyric acid type A receptor subunit beta3
		0,008	-0,42	PFDN5	Uncharacterized protein
		0,019	-0,30	GNPDA2	Glucosamine-6-phosphate isomerase
		0,024	-0,30	GOLGA3	Golgin A3
		0,025	-0,36	TXNRD2	Thioredoxin-disulfide reductase;Thioredoxin-disulfide reductase;Thioredoxin reductase

Supplementary Table S6. Cerebral proteomic profile

Group comparison	Up/Down	p-value	Log FC Difference	Genes	Protein Descriptions
					2;Thioredoxin reductase 2;Thioredoxin-disulfide reductase
		0,024	-1,04	FAM131B	Family with sequence similarity 131 member B
		0,004	-0,29	DNAJA3	DnaJ homolog subfamily A member 3, mitochondrial isoform 1;Uncharacterized protein;Uncharacterized protein
		0,038	-0,16	ADD3	Adducin 3
		0,022	-0,20	SPAG9	Sperm associated antigen 9
		0,042	-0,56	SUN1	Sad1 and UNC84 domain containing 1
		0,046	-0,26	RP2	Protein XRP2
		0,019	-0,48	TENM2	Teneurin transmembrane protein 2
		0,041	-0,69	BORCS5	BLOC-1-related complex subunit 5
		0,017	-0,70	UNC5C	Netrin receptor UNC5
		0,035	-0,28	MPI	Mannose-6-phosphate isomerase
		0,023	-0,85	YKT6	Uncharacterized protein
		0,034	-0,22	EWSR1	EWS RNA binding protein 1
		0,007	-0,54	LAMTOR5	Ragulator complex protein LAMTOR5
		0,005	-0,46	CAMK1D	Calcium/calmodulin dependent protein kinase ID
		0,015	-0,32	MAN2B1	Alpha-mannosidase
		0,007	-0,24	NSFL1C	NSFL1 cofactor p47
		0,016	-0,55	SPATS2L	Spermatogenesis associated serine rich 2 like
		0,009	-0,35	AMER2	APC membrane recruitment protein 2
		0,039	-0,28	NPTXR	Neuronal pentraxin receptor
		0,047	-1,19	GFRA1	GDNF family receptor alpha-1;GDNF family receptor alpha 1
		0,046	-0,47	PLPPR3	Phospholipid phosphatase-related protein type 3 isoform 2
		0,027	-0,23	SPTBN4	Spectrin beta, non-erythrocytic 4
		0,014	-0,75	PCDHGA4	Uncharacterized protein
		0,036	-0,25	FAM171A1	Family with sequence similarity 171 member A1
		0,020	-0,74	EPG5	Ectopic P-granules autophagy protein 5 homolog

Supplementary Table S6. Cerebral proteomic profile

Group comparison	Up/Down	p-value	Log FC Difference	Genes	Protein Descriptions
		0,034	-1,01	DDC	Aromatic-L-amino-acid decarboxylase
		0,041	-0,24	DBT	Dihydrolipoamide acetyltransferase component of pyruvate dehydrogenase complex
		0,048	-2,68	TMEM201	Transmembrane protein 201
DMF vs Sham	Upregulated	0,050	0,30	CCDC177	Coiled-coil domain containing 177
		0,033	0,63	SMIM13	Uncharacterized protein
		0,042	1,29	PRKAR2A	cAMP-dependent protein kinase type II-alpha regulatory subunit (Fragment)
		0,036	0,13	PSMD3	26S proteasome non-ATPase regulatory subunit 3
		0,045	0,70	KRT5	IF rod domain-containing protein
		0,038	0,60	SMPD4	Sphingomyelin phosphodiesterase 4
		0,022	0,95	LIN7B	Lin-7 homolog B, crumbs cell polarity complex component
		0,012	0,43	LSM4	U6 snRNA-associated Sm-like protein LSm4
		0,002	0,76	RNF126	RING-type E3 ubiquitin transferase
		0,036	0,34	COPZ1	Coatomer subunit zeta
		0,005	0,54	NUDT7	Nudix hydrolase 7
		0,049	0,34	APC2	APC regulator of WNT signaling pathway 2
		0,018	0,88	CNOT4	CCR4-NOT transcription complex subunit 4
		0,014	0,28	AKAP5	A-kinase anchoring protein 5
		0,035	0,55	AHNAK	AHNAK nucleoprotein
		0,023	3,98	NPDC1	Neural proliferation, differentiation and control 1;Neural proliferation, differentiation and control 1
		0,019	0,99	CFD	Complement factor D
		0,045	1,01	RAF1	Uncharacterized protein;Uncharacterized protein;RAF proto-oncogene serine/threonine-protein kinase isoform a
		0,045	0,44	RALBP1	RalA binding protein 1
		0,042	1,88	PEAK1	Pseudopodium enriched atypical kinase 1
		0,036	0,18	PDK3	Protein-serine/threonine kinase
		0,043	0,57	CREB1	Cyclic AMP-responsive element-binding protein 1 isoform B;cAMP responsive element binding

Supplementary Table S6. Cerebral proteomic profile

Group comparison	Up/Down	p-value	Log FC Difference	Genes	Protein Descriptions
					protein 1;cAMP responsive element binding protein 1;cAMP responsive element binding protein 1;cAMP responsive element binding protein 1
		0,025	0,32	DNAJB4	DnaJ heat shock protein family (Hsp40) member B4
		0,039	0,43	RBP1	FABP domain-containing protein
		0,018	0,98	STMN3	Stathmin
		0,015	2,13	OXSM	Beta-ketoacyl-[acyl-carrier-protein] synthase I;3-oxoacyl-[acyl-carrier-protein] synthase, mitochondrial
		0,024	0,35	FTO	Alpha-ketoglutarate-dependent dioxygenase FTO
		0,023	0,40	DPYD	Dihydropyrimidine dehydrogenase [NADP(+)]
		0,024	1,10	SENP8	SUMO peptidase family member, NEDD8 specific
		0,047	0,53	SPRED1	Sprouty related EVH1 domain containing 1
		0,002	0,50	ALDH1B1	Aldehyde dehydrogenase 1 family member B1
		0,022	0,33	XRCC6	ATP-dependent DNA helicase 2 subunit 1
		0,011	0,57	MYLK	Myosin light chain kinase, smooth muscle;Myosin light chain kinase, smooth muscle;Myosin light chain kinase, smooth muscle;Myosin light chain kinase, smooth muscle;Myosin light chain kinase, smooth muscle
		0,035	2,00	ADCY6	Adenylate cyclase type 6
		0,032	0,22	EIF3G	Eukaryotic translation initiation factor 3 subunit G
		0,033	0,42	DIAPH1	Diaphanous related formin 1
		0,042	1,03	ABLIM2	Actin binding LIM protein family member 2
		0,039	0,36	MINDY2	Ubiquitin carboxyl-terminal hydrolase
		0,016	0,32	PEX5L	Peroxisomal biogenesis factor 5 like
		0,028	0,14	NUDT21	Cleavage and polyadenylation specificity factor subunit 5
		0,047	0,30	ZC3H7B	Zinc finger CCCH domain-containing protein 7B
		0,041	0,25	PDK2	Protein-serine/threonine kinase
		0,034	0,58	PRRT3	Proline rich transmembrane protein 3

Supplementary Table S6. Cerebral proteomic profile

Group comparison	Up/Down	p-value	Log FC Difference	Genes	Protein Descriptions
		0,015	0,41	ARPC1A	Actin-related protein 2/3 complex subunit
		0,048	0,13	USP7	Ubiquitin carboxyl-terminal hydrolase 7
		0,037	0,94	RAB3IP	Sec2p domain-containing protein
		0,031	0,55	EPHB4	Receptor protein-tyrosine kinase
		0,013	1,41	GGACT	Gamma-glutamylaminecyclotransferase
		0,026	0,73	METAP1	Methionine aminopeptidase
		0,002	1,16	ALDH1A1	Aldehyde dehydrogenase 1 family member A1
		0,007	0,36	MT-ND1	NADH-ubiquinone oxidoreductase chain 1
	Downregulated	0,041	-0,50	RARS2	Arginyl-tRNA synthetase
		0,029	-0,65	MRPL47	Mitochondrial ribosomal protein L47
		0,043	-0,18	SRSF3	Serine and arginine rich splicing factor 3
		0,030	-0,40	UBE2D1;UBE2D1;UBE2D1;UBE2D4;UBE2D4;UBE2D1	Ubiquitin conjugating enzyme E2 D1;Ubiquitin conjugating enzyme E2 D1;Ubiquitin conjugating enzyme E2 D1;UBC core domain-containing protein;UBC core domain-containing protein;UB2D1
		0,038	-0,15	NF1	Neurofibromin 1
		0,010	-0,56	HYI	Putative hydroxypyruvate isomerase
		0,011	-0,55	HINT3	Histidine triad nucleotide binding protein 3
		0,016	-0,27	POR	NADPH--cytochrome P450 reductase
		0,023	-0,18	CAND2	TIP120 domain-containing protein
		0,011	-0,39	NT5C3B	5'-nucleotidase
		0,011	-0,32	GMPPB	Mannose-1-phosphate guanyltransferase beta
		0,035	-0,57	NUDT2	Bis(5'-nucleosyl)-tetraphosphatase [asymmetrical]
		0,015	-0,82	S1PR1	Sphingosine 1-phosphate receptor 1
		0,016	-0,43	GRHPR	Glyoxylate and hydroxypyruvate reductase
		0,021	-0,15	PKM	Multifunctional fusion protein
		0,008	-0,48	SNTB1	Syntrophin beta 1
		0,002	-0,28	CCAR1	Cell division cycle and apoptosis regulator 1
		0,019	-0,63	PPDPF; LOC100513894;	Uncharacterized protein;Pancreatic progenitor cell differentiation and proliferation factor homolog

Supplementary Table S6. Cerebral proteomic profile					
Group comparison	Up/Down	p-value	Log FC Difference	Genes	Protein Descriptions
		0,020	-1,05	PTGR1	15-oxoprostaglandin 13-reductase;Prostaglandin reductase 1
		0,049	-0,80	LAS1L	LAS1 like ribosome biogenesis factor
		0,016	-2,57	HP	Haptoglobin
		0,011	-0,40	SPHK2	Sphingosine kinase 2
		0,023	-0,29	AGPAT5	1-acylglycerol-3-phosphate O-acyltransferase 5
		0,017	-0,27	ZFR	Zinc finger RNA binding protein
		0,010	-0,64	C6H19orf12	Chromosome 6 C19orf12 homolog
		0,025	-0,97	FAM151B	Family with sequence similarity 151 member B
		0,049	-0,43	CTPS1	CTP synthase
		0,045	-0,28	FBL	Fibrillarin
		0,046	-0,72	GOLIM4	Golgi integral membrane protein 4
		0,028	-0,35	EEF1E1	Eukaryotic translation elongation factor 1 epsilon-1 isoform 1
		0,044	-0,70	DOCK2	Dedicator of cytokinesis 2
		0,035	-0,38	CBX5	Chromobox 5
		0,019	-0,50	UBE2F	UBC core domain-containing protein
		0,033	-0,57	AGPS	Alkylglycerone-phosphate synthase
		0,044	-0,21	ATXN10	Ataxin 10
		0,043	-0,14	DDX17	RNA helicase
		0,020	-0,34	FAM120B	Constitutive coactivator of peroxisome proliferator-activated receptor gamma isoform a
		0,004	-0,60	KSR1	Kinase suppressor of ras 1
		0,012	-1,44	STBD1	CBM20 domain-containing protein
		0,001	-0,95	VANGL2	Vang-like protein
		0,018	-1,53		Ig-like domain-containing protein
		0,042	-0,15	DOCK4	Dedicator of cytokinesis 4
		0,020	-0,98	GFRA1	GDNF family receptor alpha-1;GDNF family receptor alpha 1
		0,036	-0,72	PCDHGA4	Uncharacterized protein, Protocadherin gamma-A4
		0,037	-0,28	DNAJC19	J domain-containing protein

Supplementary Table S6. Cerebral proteomic profile					
Group comparison	Up/Down	p-value	Log FC Difference	Genes	Protein Descriptions
		0,036	-0,31	VKORC1L1	Vitamin-K-epoxide reductase (warfarin-sensitive)
		0,012	-0,41	COX20	Cytochrome c oxidase assembly protein COX20, mitochondrial
		0,023	-1,23	CSTF1	Cleavage stimulation factor 50 kDa subunit
		0,009	-0,46	ASCC2	Activating signal cointegrator 1 complex subunit 2
		0,009	-0,51	GGH	Folate gamma-glutamyl hydrolase
		0,040	-0,98	KANK1	ANK_REP_REGION domain-containing protein
		0,046	-2,08	HEBP2	Uncharacterized protein, Heme binding protein 2
		0,031	-0,28	FAM98A	Family with sequence similarity 98 member A
		0,036	-0,31	HEXA	Beta-hexosaminidase
		0,035	-0,62	SPCS2	Microsomal signal peptidase 25 kDa subunit
		0,041	-0,53	SEZ6	Seizure related 6 homolog
		0,008	-0,54	ELMOD1	ELMO domain containing 1
		0,017	-0,19	FBXO21	F-box protein 21
		0,024	-0,21	RICTOR	RPTOR independent companion of MTOR complex 2
		0,029	-0,46	RPL35	60S ribosomal protein L35
		0,041	-0,22	TNR	Tenascin R
		0,013	-0,46		Uncharacterized protein
		0,021	-1,19	GOSR1	Golgi SNAP receptor complex member 1;28 kDa Golgi SNARE protein;Golgi SNAP receptor complex member 1;28 kDa Golgi SNARE protein;Golgi SNAP receptor complex member 1

Protein expression was measured in cortex of sham (sham) and cardiac arrest animals who received either placebo (placebo) or dimethyl fumarate (DMF) treatment. A t-test was subsequently employed to identify differentially expressed proteins between placebo and shams, DMF and placebo and DMF and shams using p value<0.05 as significant threshold. FC = fold change.

Supplementary Table S7. Cerebral proteomic profile - Pathway enrichment analysis

Group comparison	Up/Down regulation	Pathway	FDR	P value	Gene ID	Gene Name
Placebo vs Sham	Down	Amino sugar and nucleotide sugar metabolism	≤0.05	4,32E-05	GMPPA	GDP-mannose pyrophosphorylase A
					GMPPB	GDP-mannose pyrophosphorylase B
					GNPDA1	glucosamine-6-phosphate deaminase 1
					GNPNAT1	glucosamine-phosphate N-acetyltransferase 1
					HEXA	hexosaminidase subunit alpha
		Metabolic pathways	≤0.05	6,77E-05	AGPAT1	1-acylglycerol-3-phosphate O-acyltransferase 1
					AGPAT5	1-acylglycerol-3-phosphate O-acyltransferase 5
					AGPS	alkylglycerone phosphate synthase
					ATP5F1E	ATP synthase F1 subunit epsilon
					CTPS1	CTP synthase 1
					DLAT	dihydrolipoamide S-acetyltransferase
					EBP	EBP, cholestenol delta-isomerase
					FDPS	farnesyl diphosphate synthase
					G6PC3	glucose-6-phosphatase catalytic subunit 3
					GMPPA	GDP-mannose pyrophosphorylase A

Supplementary Table S7. Cerebral proteomic profile - Pathway enrichment analysis

Group comparison	Up/Down regulation	Pathway	FDR	P value	Gene ID	Gene Name
					GMPPB	GDP-mannose pyrophosphorylase B
					GNPDA1	glucosamine-6-phosphate deaminase 1
					GOT2	glutamic-oxaloacetic transaminase 2
					HEXA	hexosaminidase subunit alpha
					HSD17B10	hydroxysteroid 17-beta dehydrogenase 10
					MMUT	methylmalonyl-CoA mutase
					NDUFA11	NADH:ubiquinone oxidoreductase subunit A11
					NDUFB9	NADH:ubiquinone oxidoreductase subunit B9
					NDUFS5	NADH:ubiquinone oxidoreductase subunit S5
					NDUFV1	NADH:ubiquinone oxidoreductase core subunit V1
					POMT1	protein O-mannosyltransferase 1
					PTGES3	prostaglandin E synthase 3
					UCK2	uridine-cytidine kinase 2
		Ribosome	≤0.05	6,82E-05	RPL11	ribosomal protein L11
					RPL15	ribosomal protein L15
					RPL24	ribosomal protein L24
					RPL27A	ribosomal protein L27a

Supplementary Table S7. Cerebral proteomic profile - Pathway enrichment analysis

Group comparison	Up/Down regulation	Pathway	FDR	P value	Gene ID	Gene Name
					RPS12	ribosomal protein S12
					RPS2	ribosomal protein S2
					RPS23	ribosomal protein S23
		Alzheimer disease	>0.05	2,29E-03	ATP5F1E	ATP synthase F1 subunit epsilon
					HSD17B10	hydroxysteroid 17-beta dehydrogenase 10
					NDUFA11	NADH:ubiquinone oxidoreductase subunit A11
					NDUFB9	NADH:ubiquinone oxidoreductase subunit B9
					NDUFS5	NADH:ubiquinone oxidoreductase subunit S5
					NDUFV1	NADH:ubiquinone oxidoreductase core subunit V1
		Oxidative phosphorylation	>0.05	4,20E-03	ATP5F1E	ATP synthase F1 subunit epsilon
					NDUFA11	NADH:ubiquinone oxidoreductase subunit A11
					NDUFB9	NADH:ubiquinone oxidoreductase subunit B9
					NDUFS5	NADH:ubiquinone oxidoreductase subunit S5
					NDUFV1	NADH:ubiquinone oxidoreductase core subunit V1
		Parkinson disease	>0.05	5,72E-03	ATP5F1E	ATP synthase F1 subunit epsilon

Supplementary Table S7. Cerebral proteomic profile - Pathway enrichment analysis						
Group comparison	Up/Down regulation	Pathway	FDR	P value	Gene ID	Gene Name
					NDUFA11	NADH:ubiquinone oxidoreductase subunit A11
					NDUFB9	NADH:ubiquinone oxidoreductase subunit B9
					NDUFS5	NADH:ubiquinone oxidoreductase subunit S5
					NDUFV1	NADH:ubiquinone oxidoreductase core subunit V1
		Non-alcoholic fatty liver disease (NAFLD)	>0.05	7,18E-03	AKT3	AKT serine/threonine kinase 3
					NDUFA11	NADH:ubiquinone oxidoreductase subunit A11
					NDUFB9	NADH:ubiquinone oxidoreductase subunit B9
					NDUFS5	NADH:ubiquinone oxidoreductase subunit S5
					NDUFV1	NADH:ubiquinone oxidoreductase core subunit V1
		Spliceosome	>0.05	1,74E-02	HSPA8	heat shock protein family A (Hsp70) member 8
					LSM7	LSM7 homolog, U6 small nuclear RNA and mRNA degradation associated
					SNRPF	small nuclear ribonucleoprotein polypeptide F

Supplementary Table S7. Cerebral proteomic profile - Pathway enrichment analysis

Group comparison	Up/Down regulation	Pathway	FDR	P value	Gene ID	Gene Name
					U2AF2	U2 small nuclear RNA auxiliary factor 2
		Huntington disease	>0.05	1,86E-02	ATP5F1E	ATP synthase F1 subunit epsilon
					NDUFA11	NADH:ubiquinone oxidoreductase subunit A11
					NDUFB9	NADH:ubiquinone oxidoreductase subunit B9
					NDUFS5	NADH:ubiquinone oxidoreductase subunit S5
					NDUFV1	NADH:ubiquinone oxidoreductase core subunit V1
		Fructose and mannose metabolism	>0.05	2,75E-02	GMPPA	GDP-mannose pyrophosphorylase A
					GMPPB	GDP-mannose pyrophosphorylase B
	Up	Viral carcinogenesis	>0.05	2,98E-03	CREB1	cAMP responsive element binding protein 1
					DNAJA3	DnaJ heat shock protein family (Hsp40) member A3
					HDAC6	histone deacetylase 6
					PIK3R1	phosphoinositide-3-kinase regulatory subunit 1
					USP7	ubiquitin specific peptidase 7
		Epstein-Barr virus infection	>0.05	3,75E-03	FADD	Fas associated via death domain

Supplementary Table S7. Cerebral proteomic profile - Pathway enrichment analysis

Group comparison	Up/Down regulation	Pathway	FDR	P value	Gene ID	Gene Name
					PIK3R1	phosphoinositide-3-kinase regulatory subunit 1
					PSMD1	proteasome 26S subunit, non-ATPase 1
					TAP1	transporter 1, ATP binding cassette subfamily B member
					USP7	ubiquitin specific peptidase 7
		Spliceosome	>0.05	4,34E-03	LOC102162880	pre-mRNA-splicing factor ISY1 homolog
					LSM4	LSM4 homolog, U6 small nuclear RNA and mRNA degradation associated
					SNRNP70	small nuclear ribonucleoprotein U1 subunit 70
					SRSF2	serine and arginine rich splicing factor 2
		Renin secretion	>0.05	5,21E-03	CREB1	cAMP responsive element binding protein 1
					GUCY1A1	guanylate cyclase 1 soluble subunit alpha 1
					GUCY1B1	guanylate cyclase 1 soluble subunit beta 1
		Hepatitis B	>0.05	6,19E-03	CREB1	cAMP responsive element binding protein 1
					FADD	Fas associated via death domain

Supplementary Table S7. Cerebral proteomic profile - Pathway enrichment analysis

Group comparison	Up/Down regulation	Pathway	FDR	P value	Gene ID	Gene Name
					LAMTOR5	late endosomal/lysosomal adaptor, MAPK and MTOR activator 5
					PIK3R1	phosphoinositide-3-kinase regulatory subunit 1
		Gap junction	>0.05	1,09E-02	GUCY1A1	guanylate cyclase 1 soluble subunit alpha 1
					GUCY1B1	guanylate cyclase 1 soluble subunit beta 1
					TUBB4B	tubulin beta 4B class IVb
		Herpes simplex infection	>0.05	1,39E-02	FADD	Fas associated via death domain
					SRSF2	serine and arginine rich splicing factor 2
					TAP1	transporter 1, ATP binding cassette subfamily B member
					USP7	ubiquitin specific peptidase 7
		Circadian entrainment	>0.05	1,43E-02	CREB1	cAMP responsive element binding protein 1
					GUCY1A1	guanylate cyclase 1 soluble subunit alpha 1
					GUCY1B1	guanylate cyclase 1 soluble subunit beta 1
		TNF signaling pathway	>0.05	1,88E-02	CREB1	cAMP responsive element binding protein 1
					FADD	Fas associated via death domain
					PIK3R1	phosphoinositide-3-kinase regulatory subunit 1

Supplementary Table S7. Cerebral proteomic profile - Pathway enrichment analysis

Group comparison	Up/Down regulation	Pathway	FDR	P value	Gene ID	Gene Name
		Platelet activation	>0.05	2,73E-02	GUCY1A1	guanylate cyclase 1 soluble subunit alpha 1
					GUCY1B1	guanylate cyclase 1 soluble subunit beta 1
					PIK3R1	phosphoinositide-3-kinase regulatory subunit 1
DMF vs Placebo	Down	Viral carcinogenesis	>0.05	4,28E-04	DNAJA3	DnaJ heat shock protein family (Hsp40) member A3
					GTF2H1	general transcription factor IIH subunit 1
					HDAC2	histone deacetylase 2
					NFKB1	nuclear factor kappa B subunit 1
					PIK3R1	phosphoinositide-3-kinase regulatory subunit 1
					STAT3	signal transducer and activator of transcription 3
					TBPL1	TATA-box binding protein like 1
		Cocaine addiction	>0.05	5,30E-03	DDC	dopa decarboxylase
					GPSM1	G protein signaling modulator 1
					NFKB1	nuclear factor kappa B subunit 1
		AGE-RAGE signaling pathway in diabetic complications	>0.05	6,56E-03	NFKB1	nuclear factor kappa B subunit 1
					PIK3R1	phosphoinositide-3-kinase regulatory subunit 1
					STAT3	signal transducer and activator of transcription 3

Supplementary Table S7. Cerebral proteomic profile - Pathway enrichment analysis

Group comparison	Up/Down regulation	Pathway	FDR	P value	Gene ID	Gene Name
					THBD	thrombomodulin
		Acute myeloid leukemia	>0.05	1,24E-02	NFKB1	nuclear factor kappa B subunit 1
					PIK3R1	phosphoinositide-3-kinase regulatory subunit 1
					STAT3	signal transducer and activator of transcription 3
		Prolactin signaling pathway	>0.05	1,58E-02	NFKB1	nuclear factor kappa B subunit 1
					PIK3R1	phosphoinositide-3-kinase regulatory subunit 1
					STAT3	signal transducer and activator of transcription 3
		Pancreatic cancer	>0.05	1,71E-02	NFKB1	nuclear factor kappa B subunit 1
					PIK3R1	phosphoinositide-3-kinase regulatory subunit 1
					STAT3	signal transducer and activator of transcription 3
		Fluid shear stress and atherosclerosis	>0.05	1,80E-02	NFKB1	nuclear factor kappa B subunit 1
					PIK3R1	phosphoinositide-3-kinase regulatory subunit 1
					SDC4	syndecan 4
					THBD	thrombomodulin
		Chronic myeloid leukemia	>0.05	1,83E-02	HDAC2	histone deacetylase 2
					NFKB1	nuclear factor kappa B subunit 1
					PIK3R1	phosphoinositide-3-kinase regulatory subunit 1

Supplementary Table S7. Cerebral proteomic profile - Pathway enrichment analysis						
Group comparison	Up/Down regulation	Pathway	FDR	P value	Gene ID	Gene Name
		Apoptosis	>0.05	1,84E-02	DAB2IP	DAB2 interacting protein
					LMNA	lamin A/C
					NFKB1	nuclear factor kappa B subunit 1
					PIK3R1	phosphoinositide-3-kinase regulatory subunit 1
		Hepatitis B	>0.05	1,93E-02	LAMTOR5	late endosomal/lysosomal adaptor, MAPK and MTOR activator 5
					NFKB1	nuclear factor kappa B subunit 1
					PIK3R1	phosphoinositide-3-kinase regulatory subunit 1
					STAT3	signal transducer and activator of transcription 3
	Up	Ribosome	≤0.05	1,02E-06	RPL11	ribosomal protein L11
					RPL13A	ribosomal protein L13a
					RPL27A	ribosomal protein L27a
					RPL34	ribosomal protein L34
					RPL35A	ribosomal protein L35a
					RPLP0	ribosomal protein lateral stalk subunit P0
					RPS11	ribosomal protein S11
					RPS18	ribosomal protein S18
					RPS19	ribosomal protein S19
					RPS2	ribosomal protein S2
		Glycolysis / Gluconeogenesis	≤0.05	4,31E-06	ADH5	alcohol dehydrogenase 5 (class III), chi polypeptide

Supplementary Table S7. Cerebral proteomic profile - Pathway enrichment analysis						
Group comparison	Up/Down regulation	Pathway	FDR	P value	Gene ID	Gene Name
					ALDH1B1	aldehyde dehydrogenase 1 family member B1
					ALDH2	aldehyde dehydrogenase 2 family member
					HK2	hexokinase 2
					PDHA1	pyruvate dehydrogenase E1 alpha 1 subunit
					PGK1	phosphoglycerate kinase 1
					PGM1	phosphoglucomutase 1
		Fatty acid degradation	≤0.05	4,65E-06	ACSL4	acyl-CoA synthetase long chain family member 4
					ADH5	alcohol dehydrogenase 5 (class III), chi polypeptide
					ALDH1B1	aldehyde dehydrogenase 1 family member B1
					ALDH2	aldehyde dehydrogenase 2 family member
					CPT1A	carnitine palmitoyltransferase 1A
					HADHA	hydroxyacyl-CoA dehydrogenase trifunctional multienzyme complex subunit alpha
		Valine, leucine and isoleucine degradation	≤0.05	1,97E-04	ALDH1B1	aldehyde dehydrogenase 1 family member B1
					ALDH2	aldehyde dehydrogenase 2 family member
					BCAT1	branched chain amino acid transaminase 1
					HADHA	hydroxyacyl-CoA dehydrogenase

Supplementary Table S7. Cerebral proteomic profile - Pathway enrichment analysis

Group comparison	Up/Down regulation	Pathway	FDR	P value	Gene ID	Gene Name
						trifunctional multienzyme complex subunit alpha
					HSD17B10	hydroxysteroid 17-beta dehydrogenase 10
		Huntington disease	≤0.05	2,08E-04	APAF1	apoptotic peptidase activating factor 1
					ATP5F1E	ATP synthase F1 subunit epsilon
					CLTB	clathrin light chain B
					COX6C	COX6C protein
					COX7C	cytochrome c oxidase subunit VIIc
					NDUFA11	NADH:ubiquinone oxidoreductase subunit A11
					NDUFB9	NADH:ubiquinone oxidoreductase subunit B9
					NDUFC2	NADH:ubiquinone oxidoreductase subunit C2
					SOD1	superoxide dismutase 1
		Metabolic pathways	≤0.05	2,40E-04	ACSL4	acyl-CoA synthetase long chain family member 4
					ADH5	alcohol dehydrogenase 5 (class III), chi polypeptide
					ALDH1B1	aldehyde dehydrogenase 1 family member B1
					ALDH2	aldehyde dehydrogenase 2 family member
					ATP5F1E	ATP synthase F1 subunit epsilon

Supplementary Table S7. Cerebral proteomic profile - Pathway enrichment analysis						
Group comparison	Up/Down regulation	Pathway	FDR	P value	Gene ID	Gene Name
					BCAT1	branched chain amino acid transaminase 1
					COX6C	COX6C protein
					COX7C	cytochrome c oxidase subunit VIIc
					CRYL1	crystallin lambda 1
					DHCR7	7-dehydrocholesterol reductase
					FUT9	fucosyltransferase 9
					GNPDA1	glucosamine-6-phosphate deaminase 1
					HADHA	hydroxyacyl-CoA dehydrogenase trifunctional multienzyme complex subunit alpha
					HK2	hexokinase 2
					HSD17B10	hydroxysteroid 17-beta dehydrogenase 10
					HSD17B12	hydroxysteroid 17-beta dehydrogenase 12
					LSS	lanosterol synthase
					NAMPT	nicotinamide phosphoribosyltransferase
					NDUFA11	NADH:ubiquinone oxidoreductase subunit A11
					NDUFB9	NADH:ubiquinone oxidoreductase subunit B9
					NDUFC2	NADH:ubiquinone oxidoreductase subunit C2

Supplementary Table S7. Cerebral proteomic profile - Pathway enrichment analysis

Group comparison	Up/Down regulation	Pathway	FDR	P value	Gene ID	Gene Name
					PDHA1	pyruvate dehydrogenase E1 alpha 1 subunit
					PGK1	phosphoglycerate kinase 1
					PGM1	phosphoglucomutase 1
					PPAT	phosphoribosyl pyrophosphate amidotransferase
					SMPD3	sphingomyelin phosphodiesterase 3
					UMPS	uridine monophosphate synthetase
		Alzheimer disease	≤0.05	4,61E-04	APAF1	apoptotic peptidase activating factor 1
					ATP5F1E	ATP synthase F1 subunit epsilon
					COX6C	COX6C protein
					COX7C	cytochrome c oxidase subunit VIIc
					HSD17B10	hydroxysteroid 17-beta dehydrogenase 10
					NDUFA11	NADH:ubiquinone oxidoreductase subunit A11
					NDUFB9	NADH:ubiquinone oxidoreductase subunit B9
					NDUFC2	NADH:ubiquinone oxidoreductase subunit C2
		Oxidative phosphorylation	≤0.05	5,60E-04	ATP4A	ATPase H+/K+ transporting subunit alpha
					ATP5F1E	ATP synthase F1 subunit epsilon

Supplementary Table S7. Cerebral proteomic profile - Pathway enrichment analysis						
Group comparison	Up/Down regulation	Pathway	FDR	P value	Gene ID	Gene Name
					COX6C	COX6C protein
					COX7C	cytochrome c oxidase subunit VIIc
					NDUFA11	NADH:ubiquinone oxidoreductase subunit A11
					NDUFB9	NADH:ubiquinone oxidoreductase subunit B9
					NDUFC2	NADH:ubiquinone oxidoreductase subunit C2
		Pyruvate metabolism	≤0.05	6,61E-04	ALDH1B1	aldehyde dehydrogenase 1 family member B1
					ALDH2	aldehyde dehydrogenase 2 family member
					GLO1	glyoxalase I
					PDHA1	pyruvate dehydrogenase E1 alpha 1 subunit
		Parkinson disease	≤0.05	8,65E-04	APAF1	apoptotic peptidase activating factor 1
					ATP5F1E	ATP synthase F1 subunit epsilon
					COX6C	COX6C protein
					COX7C	cytochrome c oxidase subunit VIIc
					NDUFA11	NADH:ubiquinone oxidoreductase subunit A11
					NDUFB9	NADH:ubiquinone oxidoreductase subunit B9
					NDUFC2	NADH:ubiquinone oxidoreductase subunit C2

Supplementary Table S7. Cerebral proteomic profile - Pathway enrichment analysis						
Group comparison	Up/Down regulation	Pathway	FDR	P value	Gene ID	Gene Name
DMF vs Sham	Down	Glyoxylate and dicarboxylate metabolism	>0.05	6,25E-03	GRHPR	glyoxylate and hydroxypyruvate reductase
					HYI	hydroxypyruvate isomerase (putative)
		Pyrimidine metabolism	>0.05	6,39E-03	CTPS1	CTP synthase 1
					NT5C3B	5'-nucleotidase, cytosolic IIIB
					NUDT2	nudix hydrolase 2
		Pyruvate metabolism	>0.05	9,41E-03	GRHPR	glyoxylate and hydroxypyruvate reductase
					PKM	pyruvate kinase, muscle
		Amino sugar and nucleotide sugar metabolism	>0.05	1,61E-02	GMPPB	GDP-mannose pyrophosphorylase B
					HEXA	hexosaminidase subunit alpha
		Metabolic pathways	>0.05	2,02E-02	AGPAT5	1-acylglycerol-3-phosphate O-acyltransferase 5
					AGPS	alkylglycerone phosphate synthase
					CTPS1	CTP synthase 1
					GMPPB	GDP-mannose pyrophosphorylase B
					GRHPR	glyoxylate and hydroxypyruvate reductase
					HEXA	hexosaminidase subunit alpha
					HYI	hydroxypyruvate isomerase (putative)

Supplementary Table S7. Cerebral proteomic profile - Pathway enrichment analysis

Group comparison	Up/Down regulation	Pathway	FDR	P value	Gene ID	Gene Name
					NT5C3B	5'-nucleotidase, cytosolic IIIB
					PKM	pyruvate kinase, muscle
					SPHK2	sphingosine kinase 2
		Purine metabolism	>0.05	3,00E-02	NT5C3B	5'-nucleotidase, cytosolic IIIB
					NUDT2	nudix hydrolase 2
					PKM	pyruvate kinase, muscle
		Fc gamma R-mediated phagocytosis	>0.05	4,11E-02	DOCK2	dedicator of cytokinesis 2
					SPHK2	sphingosine kinase 2
		Ubiquinone and other terpenoid-quinone biosynthesis	>0.05	4,31E-02	KORC1L1	vitamin K epoxide reductase complex subunit 1 like 1
		Glycosphingolipid biosynthesis	>0.05	5,83E-02	HEXA	hexosaminidase subunit alpha
	Up	Regulation of actin cytoskeleton	>0.05	3,72E-03	APC2	APC2, WNT signaling pathway regulator
					ARPC1A	actin related protein 2/3 complex subunit 1A
					DIAPH1	diaphanous related formin 1
					MYLK	myosin light chain kinase
		beta-Alanine metabolism	>0.05	4,30E-03	ALDH1B1	aldehyde dehydrogenase 1 family member B1
					DPYD	dihydropyrimidine dehydrogenase
			>0.05	8,15E-03	ADCY6	adenylate cyclase 6

Supplementary Table S7. Cerebral proteomic profile - Pathway enrichment analysis						
Group comparison	Up/Down regulation	Pathway	FDR	P value	Gene ID	Gene Name
		Vasopressin-regulated water reabsorption			CREB1	cAMP responsive element binding protein 1
		Cushing syndrome	>0.05	1,14E-02	ADCY6	adenylate cyclase 6
					APC2	APC2, WNT signaling pathway regulator
					CREB1	cAMP responsive element binding protein 1
		cGMP-PKG signaling pathway	>0.05	1,43E-02	ADCY6	adenylate cyclase 6
					CREB1	cAMP responsive element binding protein 1
					MYLK	myosin light chain kinase
		Cortisol synthesis and secretion	>0.05	1,59E-02	ADCY6	adenylate cyclase 6
					CREB1	cAMP responsive element binding protein 1
		Renin secretion	>0.05	1,80E-02	ADCY6	adenylate cyclase 6
					CREB1	cAMP responsive element binding protein 1
		Thyroid hormone synthesis	>0.05	2,12E-02	ADCY6	adenylate cyclase 6
					CREB1	cAMP responsive element binding protein 1
		Gastric acid secretion	>0.05	2,18E-02	ADCY6	adenylate cyclase 6
					MYLK	myosin light chain kinase
		RNA degradation	>0.05	2,53E-02	CNOT4	CCR4-NOT transcription complex subunit 4
					LSM4	LSM4 homolog, U6 small nuclear RNA and mRNA degradation associated

Protein expression was measured in cortex of sham (sham) and cardiac arrest animals who received either placebo (placebo) or dimethyl fumarate (DMF) treatment. Pathway enrichment analysis performed using KEGG is shown. FC = fold change; FDR = false discovery rate. n=5.

Supplementary Figures

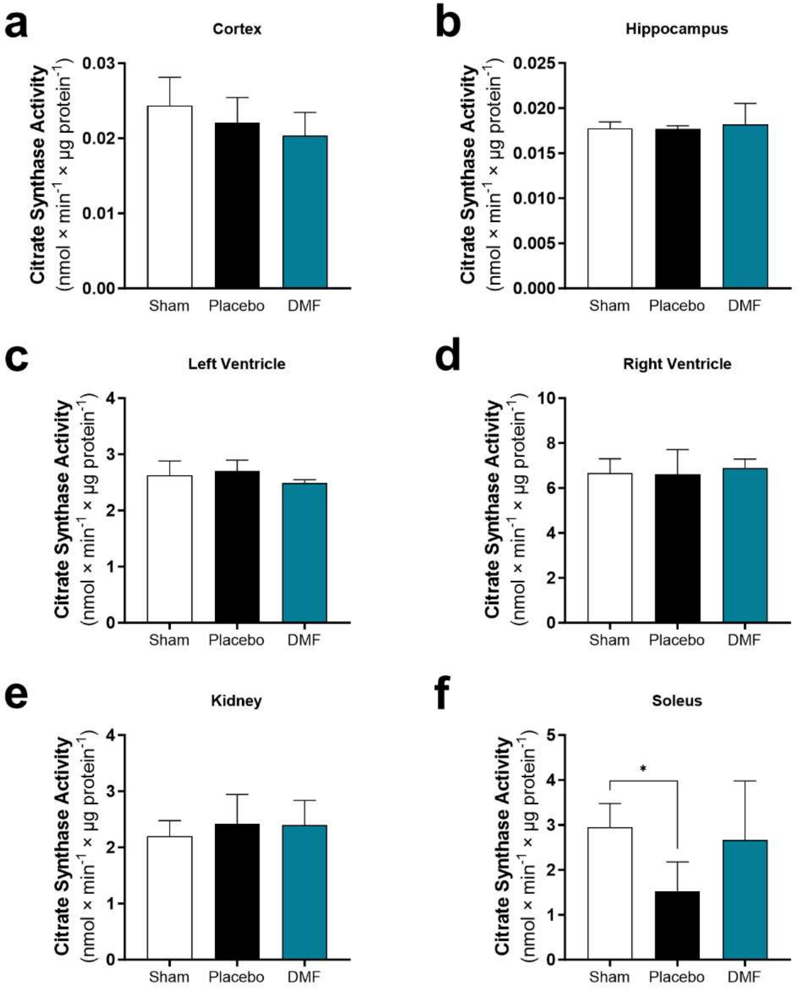

Supplementary Figure S1 Citrate synthase activity. Citrate synthase (CS) activity (nmol × min^{-1} × µg protein^{-1}) was measured as surrogate marker of mitochondrial content in (**a**) cortex, (**b**) hippocampus, (**c**) left ventricle, (**d**) right ventricle, (**e**) kidney and (**f**) soleus muscle of sham (Sham) and cardiac arrest animals who received either placebo (Placebo) or dimethyl fumarate (DMF) treatment. Normally distributed data were statistically analyzed using ordinary one-way ANOVA (homogenous variances) or Brown-Forsythe and Welch ANOVA (non-homogenous variances). For comparisons of non-normally distributed data, Kruskal-Wallis test was applied. Every group was compared to every other group to evaluate the treatment effect of DMF. *=$p < 0.05$. Data are presented as mean ± SD. n=5.

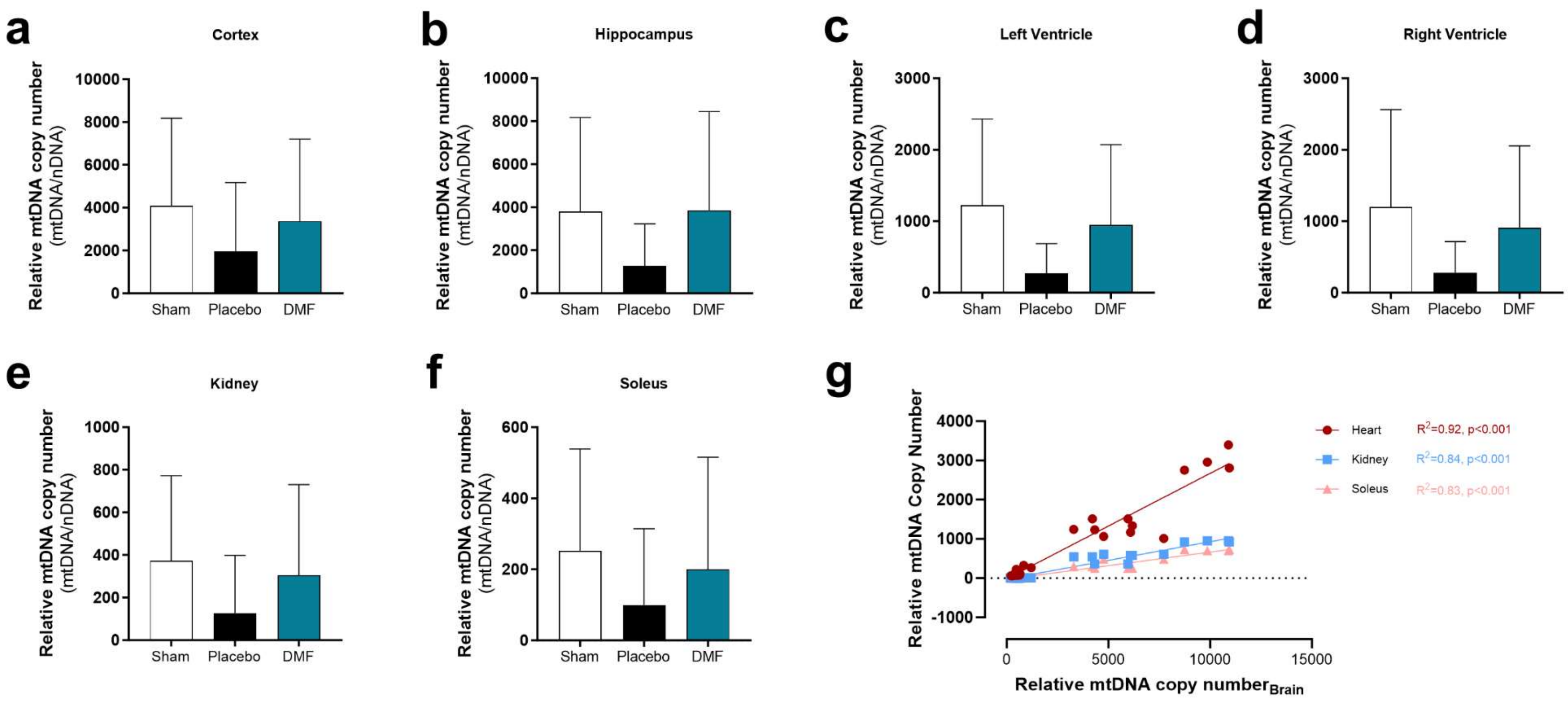

Supplementary Figure S2 Relative mtDNA copy number. Relative mtDNA copy number (mtDNA/nDNA) was measured in brain, heart, kidney and soleus muscle of sham animals (Sham) and cardiac arrest animals who received either placebo (Placebo) or dimethyl fumarate (DMF) treatment. It was determined by calculating the difference in cycle threshold (ΔC_t) between mtDNA gene copy number (MT-ND4) and the internal control of nuclear DNA (ACTB). mtDNA/nDNA of (**a**) cortex, (**b**) hippocampus, (**c**) left ventricle, (**d**) the right ventricle, (**e**) kidney and (**f**) soleus muscle is shown. Normally distributed data were statistically analyzed using ordinary one-way ANOVA (homogenous variances) or Brown-Forsythe and Welch ANOVA (non-homogenous variances). For comparisons of non-normally distributed data, Kruskal-Wallis test was applied. Every group was compared to every other group. (**g**) We further performed correlation analysis using Spearman's correlation coefficient. A correlation coefficient (R^2) of 0.1-0.3 indicated a small correlation, 0.3-0.5 indicated a moderate correlation and 0.5-0.8 indicated a strong correlation. A p value of smaller than 0.05 was considered to indicate significant difference. Data are presented as mean ± SD. n=5.

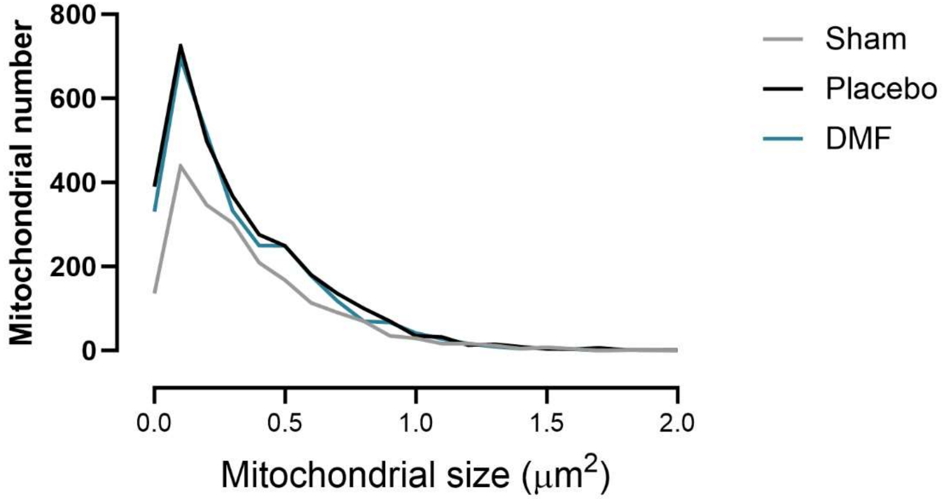

Supplementary Figure S3 Mitochondrial size distribution. We evaluated mitochondrial content and morphology using transmission electron microscopy of the left ventricular myocardium. Ultrastructural image analysis allowed assessment of mitochondrial size (μm^2) and number. Mitochondrial size distribution is shown for sham animals (Sham, grey) and cardiac arrest animals who received either placebo (Placebo, black) or dimethyl fumarate (DMF, turquoise) treatment. Data are presented as mean ± SD.

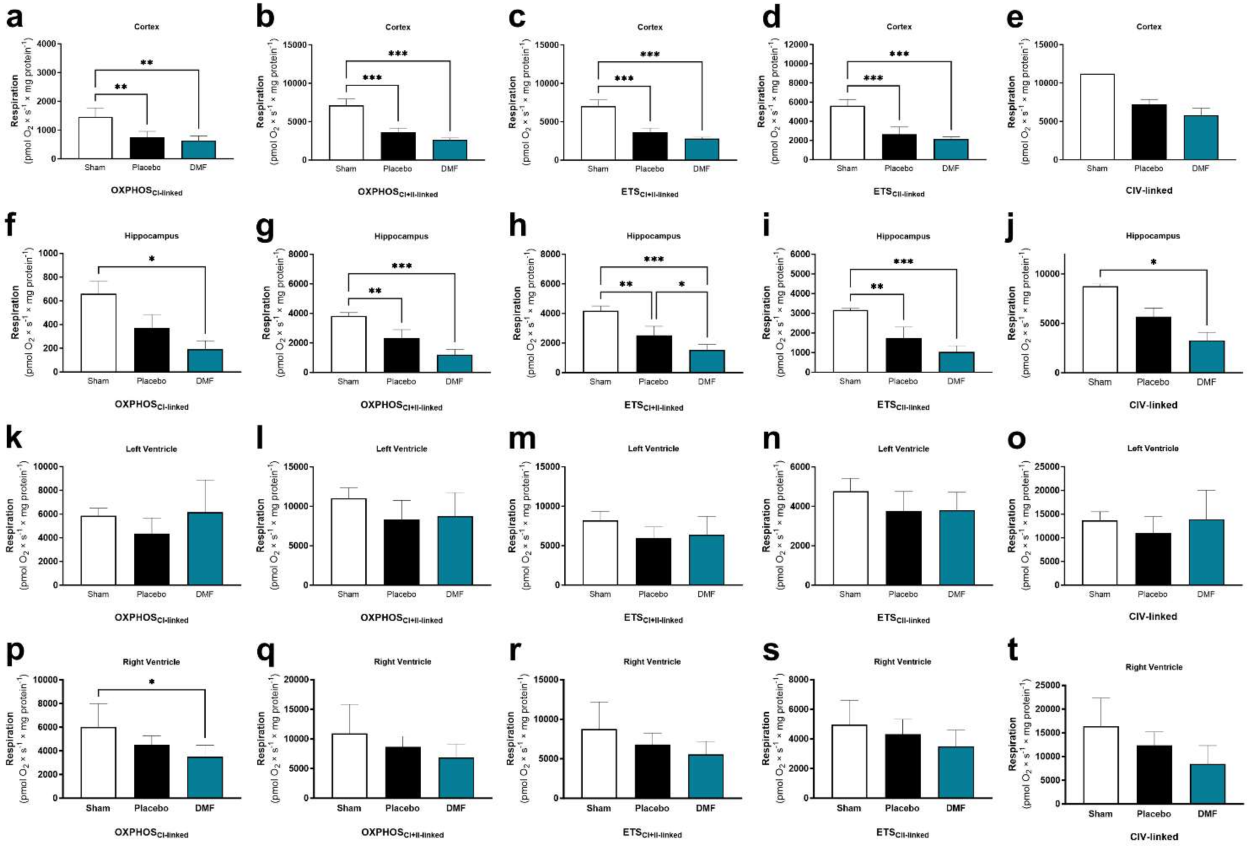

Supplementary Figure S4 Cerebral and myocardial mitochondrial respiration. Mitochondrial respiration of isolated mitochondria of cerebral cortex and hippocampus (25 µg/ml) as well as left and right ventricle (16 µg/ml) was assessed in sham animals (Sham) and cardiac arrest animals who received either placebo (Placebo) or dimethyl fumarate (DMF) treatment using high-resolution respirometry. In-depth characterization of mitochondrial respiration was performed using mitochondrial complex specific substrates and the application of a Substrate-Uncoupler-Inhibitor Titration protocol. (**a**)(**f**)(**k**)(**p**) Maximal complex I-linked and (**b**)(**g**)(**l**)(**q**) complex I+II-linked oxidative phosphorylation capacity (OXPHOS) as well as electron transport system (ETS) capacity supported by (**c**)(**h**)(**m**)(**r**) complex I+II-linked, (**d**)(**i**)(**n**)(**s**) complex II-linked and (**e**)(**j**)(**o**)(**t**) complex CIV-linked substrates is shown. Respiration is expressed as pmol $O_2 \times s^{-1} \times$ mg protein^{-1}. Normally distributed data were statistically analyzed using ordinary one-way ANOVA (homogenous variances) or Brown-Forsythe and Welch ANOVA (non-homogenous variances). For comparisons of non-normally distributed data, Kruskal-Wallis test was applied. Every group was compared to every other group. Data are presented as mean ± SD. n=5. *p<0.05, **p<0.01 and ***p<0.001. Definition of abbreviations: CI = complex I; CII = complex II; CI+II = convergent complex I and II; CIV = complex IV.

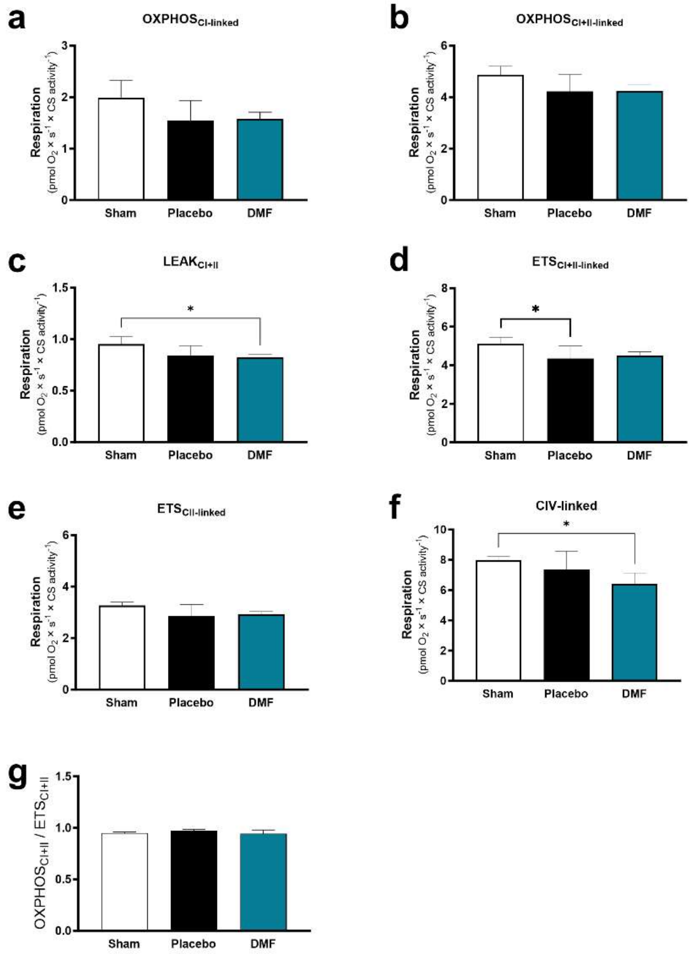

Supplementary Figure S5 Mitochondrial respiration of tissue homogenates of the cortex. Mitochondrial respiration of cortical homogenates (1 mg/mL) was assessed using high-resolution respirometry and a Substrate-Uncoupler-Inhibitor Titration protocol. (**a**) Maximal complex I-linked and (**b**) complex I + II -linked oxidative phosphorylation (OXPHOS) capacity, (**c**) $LEAK_{CI+II}$, a respiratory state where oxygen consumption is compensating for the back-flux of protons across the mitochondrial membrane, as well as electron transport system (ETS) capacity supported by (**d**) complex I and II-linked, (**e**) complex II-linked and (**f**) complex CIV-linked substrates is shown. (**g**) Additionally, the phosphorylation system control ratio ($OXPHOS_{CI+II}/ETS_{CI+II}$) was calculated to further inform whether CV-linked metabolism imposes any limitation on the rate of the ETS. Respiration is expressed as pmol $O_2 \times s^{-1} \times$ CS activity^{-1}. Normally distributed data were statistically analyzed using ordinary one-way ANOVA (homogenous variances) or Brown-Forsythe and Welch ANOVA (non-homogenous variances). For comparisons of non-normally distributed data, Kruskal-Wallis test was applied. Every group was compared to every other group. *=$p < 0.05$. Data are presented as mean ± SD. n=5. Definition of abbreviations: CI = complex I; CII = complex II; CI+II = convergent complex I and II; CIV = complex IV.

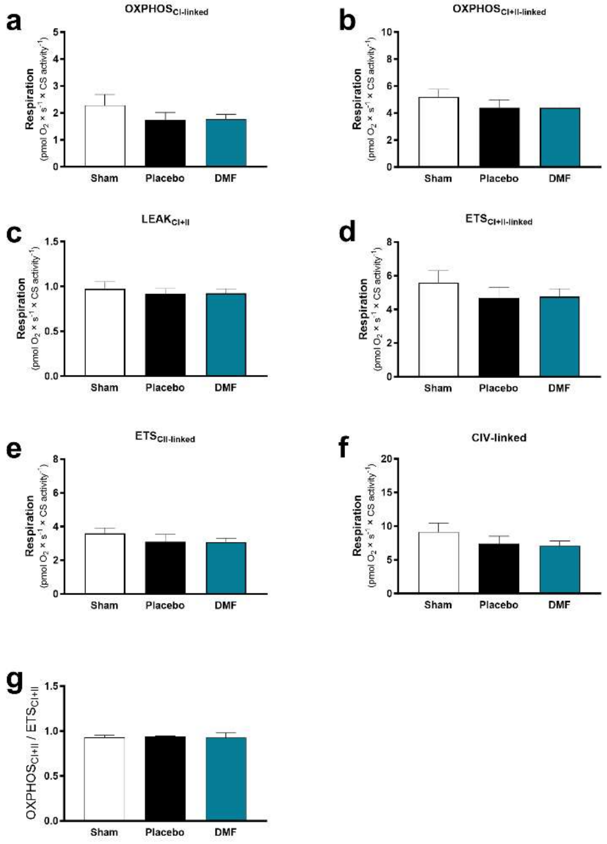

Supplementary Figure S6 Mitochondrial respiration of tissue homogenates of the hippocampus. Mitochondrial respiration of hippocampal homogenates (1 mg/mL) was assessed using high-resolution respirometry and a Substrate-Uncoupler-Inhibitor-Titration protocol. (**a**) Maximal complex I-linked and (**b**) complex I + II -linked oxidative phosphorylation (OXPHOS) capacity, (**c**) $LEAK_{CI+II}$, a respiratory state where oxygen consumption is compensating for the back-flux of protons across the mitochondrial membrane, as well as electron transport system (ETS) capacity supported by (**d**) complex I and II-linked, (**e**) complex II-linked and (**f**) complex CIV-linked substrates is shown. (**g**) Additionally, the phosphorylation system control ratio ($OXPHOS_{CI+II}/ETS_{CI+II}$) was calculated to further inform whether CV-linked metabolism imposes any limitation on the rate of the ETS. Respiration is expressed as pmol $O_2 \times s^{-1} \times$ CS activity^{-1}. Normally distributed data were statistically analyzed using ordinary one-way ANOVA (homogenous variances) or Brown-Forsythe and Welch ANOVA (non-homogenous variances). For comparisons of non-normally distributed data, Kruskal-Wallis test was applied. Every group was compared to every other group. No significant reduction in mitochondrial respiration was detected. Data are presented as mean ± SD. n=5. Definition of abbreviations: CI = complex I; CII = complex II; CI+II = convergent complex I and II; CIV = complex IV.

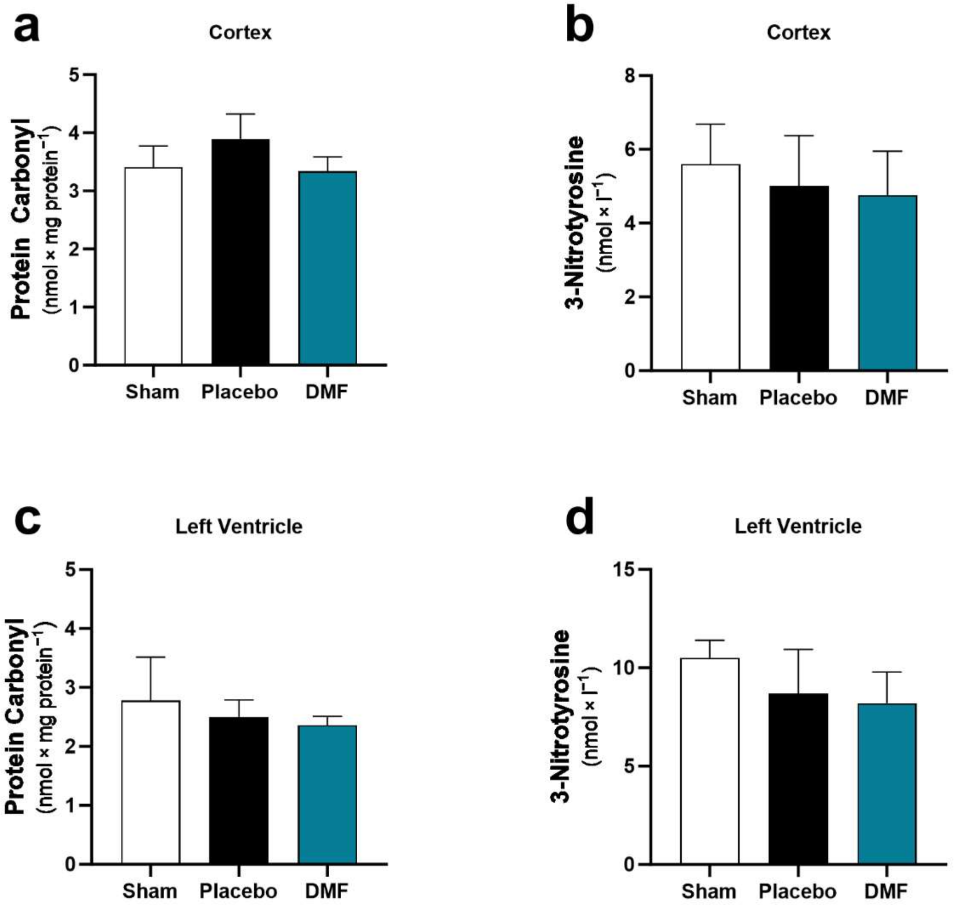

Supplementary Figure S7 Oxidative damage. Protein carbonyl and 3-nitrotyrosine oxidation was measured in the cortex and left ventricle of sham animals (Sham) and cardiac arrest animals who received either placebo (Placebo) or dimethyl fumarate (DMF). **(a)(c)** Protein carbonyl and **(b)(d)** 3-nitrotyrosine levels are shown. Protein carbonyl levels are expressed as nmol × mg protein^{-1}. 3-Nitrotyrosine levels are expressed as nmol × l^{-1}. Normally distributed data were statistically analyzed using ordinary one-way ANOVA (homogenous variances) or Brown-Forsythe and Welch ANOVA (non-homogenous variances). For comparisons of non-normally distributed data, Kruskal-Wallis test was applied. Every group was compared to every other group. No significant differences were detected. Data are presented as mean ± SD. n=5.
